# High-throughput proteome analysis reveals targeted TRPM8 degradation in prostate cancer

**DOI:** 10.18632/oncotarget.14178

**Published:** 2016-12-26

**Authors:** Swapna Asuthkar, Lusine Demirkhanyan, Samuel Robert Mueting, Alejandro Cohen, Eleonora Zakharian

**Affiliations:** ^1^ University of Illinois College of Medicine, Department of Cancer Biology and Pharmacology, Peoria, IL 61605, USA; ^2^ Proteomics and Mass Spectrometry Core Facility, Life Sciences Research Institute, Dalhousie University, Halifax, NS B3H 4R2, Canada

**Keywords:** transient receptor potential melastatin 8 channel (TRPM8), androgens, testosterone, prostate cancer, protein degradation

## Abstract

The Ca^2+^-permeable ion channel TRPM8 is a hallmark of the prostate epithelium. We recently discovered that TRPM8 is an ionotropic testosterone receptor. This finding suggested that testosterone-induced TRPM8 activity regulates Ca^2+^ homeostasis in the prostate epithelium. Since androgens are significantly implicated in prostate cancer development, the role of the novel testosterone receptor TRPM8 in cancer was assessed in our study. Although TRPM8 mRNA levels increase at the early prostate cancer stages, we found that it is not proportionally translated into TRPM8 protein levels. High-throughput proteome analysis revealed that TRPM8 degradation is enhanced in human prostate cancer cells. This degradation is executed *via* a dual degradation mechanism with the involvement of both lysosomal and proteasomal proteolytic pathways. The evaluation of the TRPM8 expression pattern in prostate cancer patients further confirmed the incidence of TRPM8 removal from the plasma membrane and its internalization pattern coincided with the severity of the tumor. Together, our results indicate that enhanced TRPM8 hydrolysis in prostate cancer could present an adaptation mechanism, sustained *via* bypassing testosterone-induced rapid Ca^2+^ uptake through TRPM8, thus, diminishing the rates of apoptosis. In this light, recovery of TRPM8 may pose a novel therapeutic strategy for an anti-tumor defense mechanism.

## INTRODUCTION

Prostate cancer (PC) is one of the devastating cancer threats to male population [1]. Currently, available treatment options have not significantly helped reduce the mortality rates from PC. Roles of androgens in genomic stimulation of prostate tissues have been well elucidated, and androgen-ablation therapy is the primary option for treating PC patients [2]. However, there is a fundamental gap in understanding the consequences of non-genomic actions exerted by androgens, testosterone in particular. Indeed, the importance of non-genomic effects of androgens has been recognized for various tissues [3]. However, the role of androgen-induced rapid signaling in cancers has received little attention. We recently made the significant breakthrough discovery of a novel testosterone receptor responsible for rapid testosterone responses with an ionotropic effect [4, 5]. This receptor is a hallmark Ca^2+^ channel of the prostate epithelium the Transient Receptor Potential Melastatin family ion channel, TRPM8 [6, 7]. We showed that picomolar concentrations of testosterone evoke rapid Ca^2+^-uptake through TRPM8 [4, 5]. This mechanism is essential for proper Ca^2+^-homeostasis and controlling cell cycle. Using planar lipid bilayers, we confirmed that this effect of testosterone on TRPM8 is direct and is not conditioned by intermediate signaling mechanisms [5].

Although, TRPM8 mRNA levels are increased during PC progression [6], this tendency is not proportionally translated to the TRPM8 protein levels. Our recent discovery showed that TRPM8 is aggressively targeted for degradation in PC, while recovery of the protein effectively suppressed tumor cells growth [8]. Together our studies suggested that TRPM8 is a key element of the orphan pathway of non-genomic testosterone-induced responses, and its activity may significantly contribute to anti-tumor defense mechanism and could serve as a novel therapeutic target. To establish the particular pathway of PC-specific TRPM8 hydrolysis here we performed high-throughput proteomic analysis and identified that PC cells enable dual-pathway degradation mechanism to eliminate TRPM8 from the plasma membrane. Furthermore, the TRPM8 protein degradation was also evident in the tissues of PC patients, confirming that it is a characteristic phenomenon common to prostate cancer progression. Establishing the precise mechanism of TRPM8 protein hydrolysis and identifying the routes for its recovery in PC cells may enable novel and specific tools to fight prostate cancer.

## RESULTS

### TRPM8 internalization correlates with the severity of prostate cancer

**Figure 1:**
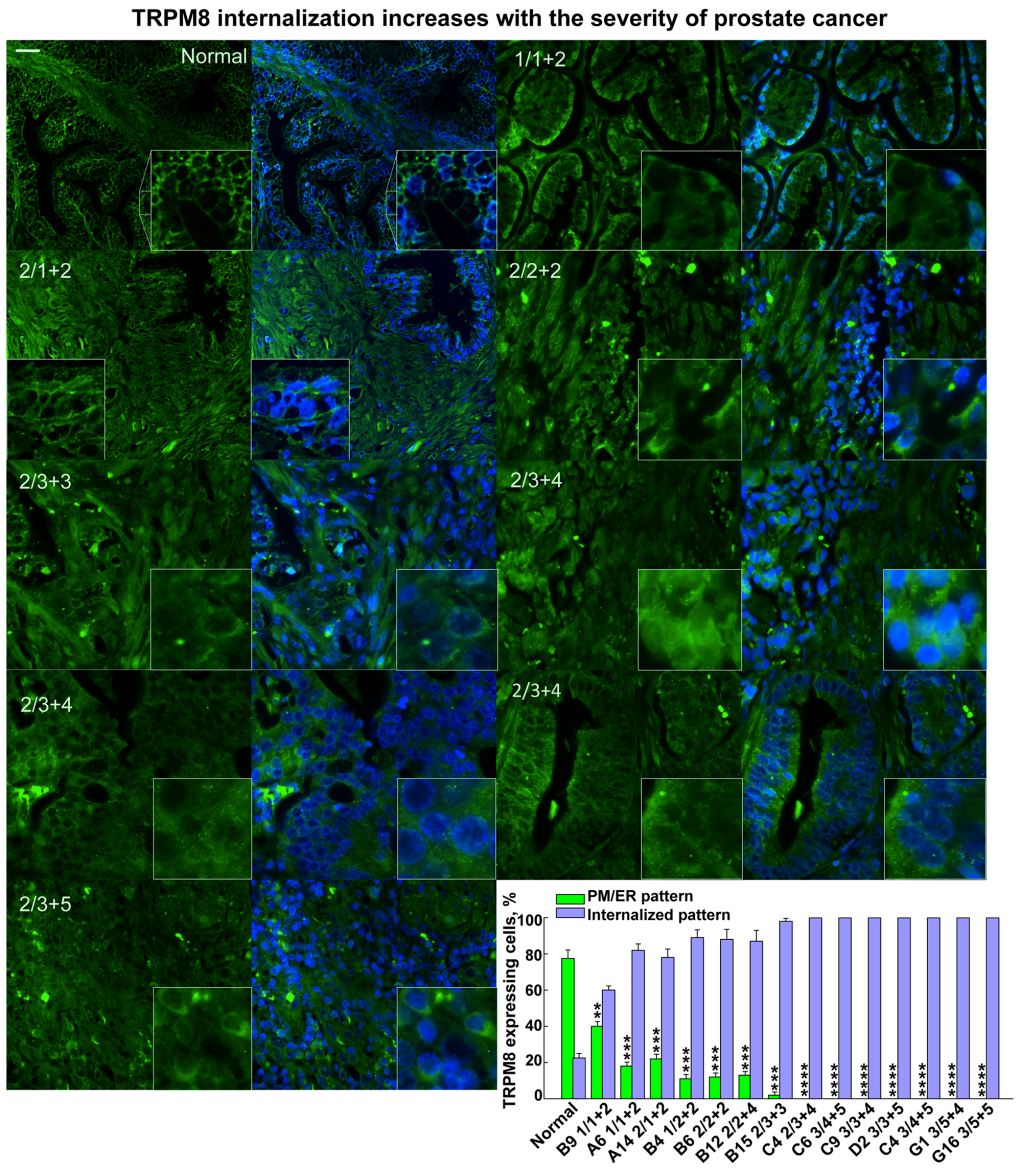
The degree of the TRPM8 protein internalization increases with severity of the human prostate cancer Immunohistochemistry analysis of a prostate cancer tissue microarray containing 60 cases (180 cores) of prostate adenocarcinoma (grades 1-4) and 9 cases (27 cores) of normal prostate tissues using anti-TRPM8 and secondary antibodies visualized with Alexa fluor-488 (green) and DAPI for the nuclear staining, as described in the Methods. The specificity of anti-TRPM8 antibodies was assessed before processing microarray and is illustrated on the WB in Supplementary Figure 1. Microscopic examination was performed (Olympus-BX61 confocal microscope) at 40X magnification. Panels demonstrate the immunohistochemical detection of the TRPM8 protein obtained from the tissue array. The scale bar is 20 μm. The numbers on the panel indicate the grade and Gleason score according to those provided by the pathology diagnosis from US Biomax, Inc. The distinct pattern of TRPM8 PM/ER localization versus internalization has been assessed using ImageJ cell count. The averaged ratios were obtained from at least 4-7 fields taken for each patient sample. The patterns as a percentile distribution are presented on the graph.

Our recent findings indicated that the hallmark Ca^2+^ channel of the prostate epithelium TRPM8 undergoes targeted degradation in the prostate cancer cell line, LNCaP [8]. Recovery of the TRPM8 protein expression on the plasma membrane significantly induced the apoptotic cell death, suggesting that this channel plays a tumor suppressor role in LNCaP cells [8]. Considering that TRPM8 might play a similar protective role in the human prostate, here we investigated the TRPM8 expression pattern in the tissues from the prostate cancer patients. Remarkably, we detected that TRPM8 expression profile is strongly altered in the tumor tissues in comparison to the healthy individuals. In line with the previous findings [7], we found that TRPM8 expression pattern was preferentially localized to the plasma membrane (PM) and endoplasmic reticulum (ER) in the normal prostate tissues. However, with cancer progression, the pattern of the protein demonstrated a significant trend for the protein internalization (Figure 1, Supplementary Figure 1, S2A-S2S). The specificity of protein immunoreactivity depends greatly on the quality and specificity of the antibodies. To ensure that the signal is TRPM8 specific, before the experiments we rigorously evaluated TRPM8 antibodies used in our study. This precaution was applied to every new batch of the antibodies obtained. To accomplish this analysis, we performed sets of controls verifying the specificity of antibodies in Western blots and immunocytochemistry. Using Western blot, we found that TRPM8 antibody revealed TRPM8 at the molecular mass of its monomer (~130 kDa), and produced similar reactivity bands compared those with anti-Myc antibodies that interact with the Myc tag at the N-terminus of TRPM8 (Supplementary Figure 1A). Staining TRPM8 in HEK-293 cells stably expressing the channel also showed the identical pattern and the characteristic presence of TRPM8 on the PM and ER (Supplementary Figure 1B). As a negative control, we tested the immunoreactivity using secondary antibodies alone, which showed no signal (Supplementary Figure 1B).

**Figure 2:**
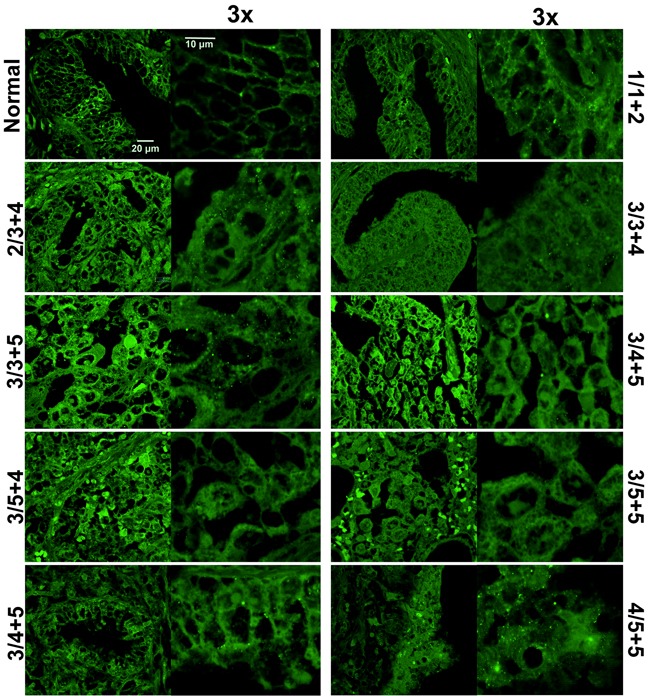
TRPM8 localization is altered in prostate cancer tissues Immunohistochemistry analysis of a prostate cancer tissue microarray demonstrates altered TRPM8 pattern in healthy and diseased tissues. The conditions are the same as described in the legend to Figure 1. The images were taken with Olympus-BX61 confocal microscope at 60X magnification, and further magnified 3x. Healthy tissues showed primary plasma membrane and endoplasmic reticulum TRPM8 localization, while this localization pattern was strongly altered in cancer tissues.

While visualizing TRPM8 expression patterns in the prostate tissue array, we found that the degree of TRPM8 internalization increased gradually along with the severity of the prostate cancer, which was assessed by the Gleason score value (Figure 1, Supplementary Figure 2A-2S). The high magnification images of intracellular TRPM8 distribution are shown in Figure 2. To validate the distinctive TRPM8 localization pattern in multiple tumor tissues we demonstrate representative histology images shown in Supplementary Figure 2A-2S. These results indicate that TRPM8 degradation is a characteristic and a common development in prostate cancer patients. They also suggest that LNCaP cells resemble the prostate cancer-specific TRPM8 phenotype and can be further used to reveal the exact mechanism of TRPM8 proteolysis.

### Ubiquitination cascade is an initial step in targeted TRPM8 proteolysis

**Figure 3:**
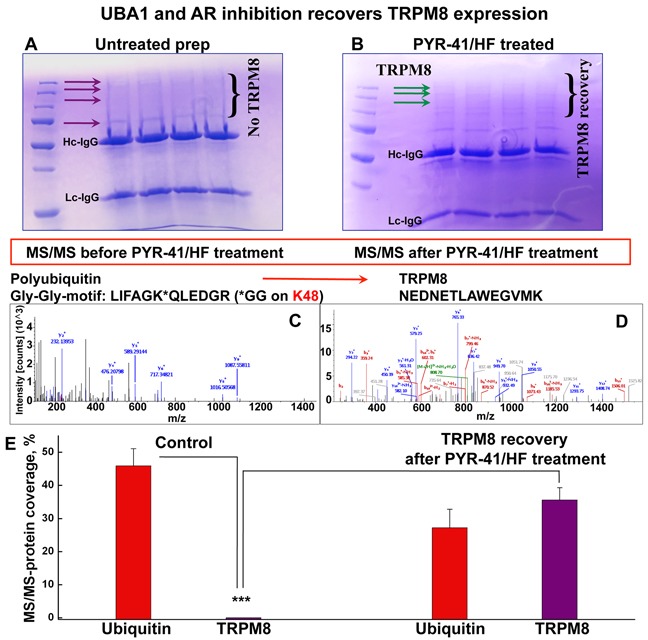
PYR-41/HF treatment recovers TRPM8 detectability in LC-MS/MS **A**. SDS-PAGE shows the absence of full-length TRPM8 IP-ed from the control LNCaP cells assessed by Coomassie staining. TRPM8 is a homotetramer and regularly migrates in the SDS gel at the apparent molecular mass of its monomer ~130 kDa, and in some conditions, depending on detergent, in the form of dimers [4]. No bands corresponding to the full-length TRPM8 were obtained for TRPM8 IP-ed from the control LNCaP. **B**. The treatment of LNCaP cells with PYR-41 (50 μM) and hydroxyflutamide (HF, 1 μM) indicated the presence of TRPM8 on the gel. **C, D**. The absence of TRPM8 in untreated control LNCaP cells and its appearance after the PYR-41/HF treatment is confirmed by LC-MS/MS. y- and b- peptide fragments ions are labeled in blue and red, respectively. All the bands obtained from the gel in panel A were screened and no TRPM8 was detected. The excised from the gel region of ~130 kDa (corresponding to TRPM8 monomer) indicated the presence of ubiquitin at high abundance levels. The MS analysis revealed a polyubiquitination site located at lysine 48 of ubiquitin. TRPM8 was detected in LC-MS/MS only after the treatment with PYR-41/HF, panel **B**. Panel **E**. demonstrates ubiquitin and TRPM8 abundance before and after the PYR-41/HF treatment (n = 9).

To obtain the details of the TRPM8 protein hydrolysis we performed liquid chromatography mass spectrometric (LC-MS/MS) analysis of TRPM8 immuno-precipitated (IP-ed) from LNCaP cells. Consistently with our previous work [8], the proteomic analysis revealed the presence of an initial enzyme of the ubiquitination cascade, UBA1. The UBA1 protein, also called E1, is the ubiquitin-activating enzyme, which requires ATP to form a thioester linkage to ubiquitin [9]. Disabling UBA1 and its downstream ubiquitin activation, we aimed mass spectrometric analysis of TRPM8 upon inhibition of UBA1 with its potent inhibitor, PYR-41. Along with PYR-41, we used an AR inhibitor, hydroxyflutamide (HF), due to the TRPM8-destabilizing effect of AR, observed in our previous work [4]. Interestingly, in the control experiments precipitating endogenous TRPM8 was inefficient, as the band corresponding to the full length of the TRPM8 monomer was not evident on the gel, neither was it detected by LC-MS/MS. However, after the cells were incubated with PYR-41/HF, the treatment resulted in the recovery of the TRPM8 monomer band evident on the gel, and the protein recovery also became detectable in the LC-MS/MS analysis (Figure 3A-3D, Supplementary Figure 3A-3H).

Although the full-length TRPM8 protein was absent in the control probes, the presence of ubiquitin was obtained at high-abundance level (Figure 3C, 3E, Supplementary Figure 3A, 3B). In contrast, the inhibition of UBA1 resulted in the recovery of TRPM8, evident both on the gel as well as the proteomic analysis (Figure 3B, 3D, 3E, Supplementary Figure 3C-3H). The details of this analysis before and after the drug treatment and the resulted specific protein hits are shown in Supplementary Figure 3A-3H.

It is critical that mass spectrometry analysis revealed the appearance of ubiquitin in its polymeric form, with the polyubiquitination site located at the lysine residue 48 (Lys48). It is important to note, that the Lys48-linked polyubiquitin is involved in the protein degradation *via* proteasome [9, 10]. Together these results indicate that TRPM8 is targeted for degradation by ubiquitin, most likely *via* the proteasomal proteolytic machinery, and upon inhibition of the initial enzyme in the ubiquitination cascade, a relative TRPM8 recovery could be achieved.

### TRPM8 undergoes ubiquitination with subsequent lysosomal and proteasomal degradation

**Figure 4:**
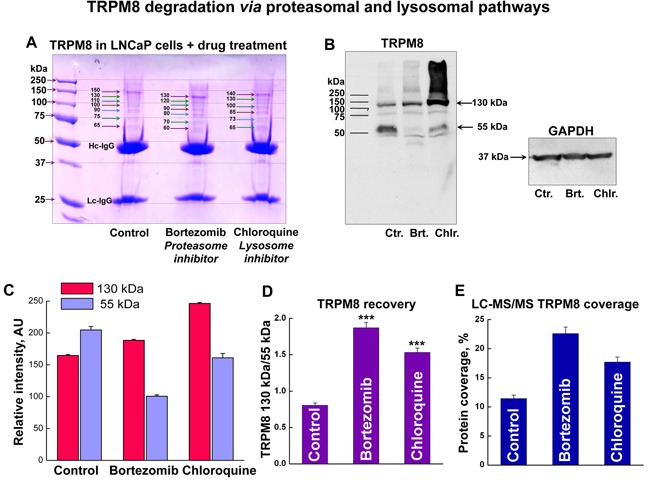
TRPM8 undergoes degradation in LNCaP cells *via* proteasomal and lysosomal pathways **A**. To enhance TRPM8 expression levels we double-transfected LNCaP cells with the Myc-tagged TRPM8, which was then IP-ed with highly specific anti-Myc-antibodies. The Coomassie stained SDS-gel shows myc-TRPM8 IP from the control LNCaP cells, and cells treated with the proteasome inhibitor bortezomib (100 nM), and the lysosome inhibitor chloroquine (200 μM) for 24 h. **B**. Western blot performed with anti-Myc-antibodies shows bands corresponding to TRPM8 monomer at ~130 kDa and degradation product at ~55 kDa. **C**. Relative intensities of the corresponding bands obtained on the WB show that while chloroquine treatment results in the total increase of ~130 kDa band, the bortezomib treatment results in the reduction of ~55 kDa band. **D**. The ratios of 130 and 55 kDa bands demonstrate the distribution of TRPM8 recovery after the drug treatment. **E**. Similar protein distribution is obtained in the relative TRPM8 protein abundance levels in LC-MS/MS analysis.

To establish the TRPM8 breakdown pathways, we next performed high-throughput proteomic analysis of TRPM8 IP-ed from LNCaP cells along with treating the cells with either lysosome or proteasome inhibitors. To increase the pool of TRPM8 and its interacting partners, we double-transfected LNCaP cells with the Myc-tagged TRPM8 construct. Enhanced expression of TRPM8, along with the immuno-precipitation using highly selective Myc-antibody, resulted in obtaining accurate proteomic profiles. All the protein bands obtained from the Coomassie-stained gels were excised and processed for mass spectrometry (Figure 4A). Interestingly, the Western blot (WB) analysis revealed that total TRPM8 expression increases upon inhibition of the lysosome-mediated degradation using lysosome inhibitor chloroquine. However, the degradation product at ~55 kDa, similar to that of control, is yet to be observed with the chloroquine treatment (Figure 4B). In contrast, inhibition of the proteasomal degradation with bortezomib resulted in substantial reduction of the ~55 kDa band. Thus, the ratio of the total TRPM8 protein recovery was greater with the bortezomib treatment (Figure 4B-4D). Remarkably, the TRPM8 protein recovery obtained in LC-MS/MS exhibited a similar pattern, with a slight prevalence upon the proteasome inhibition (Figure 4E, Supplementary Figure 4A-4I).

**Figure 5:**
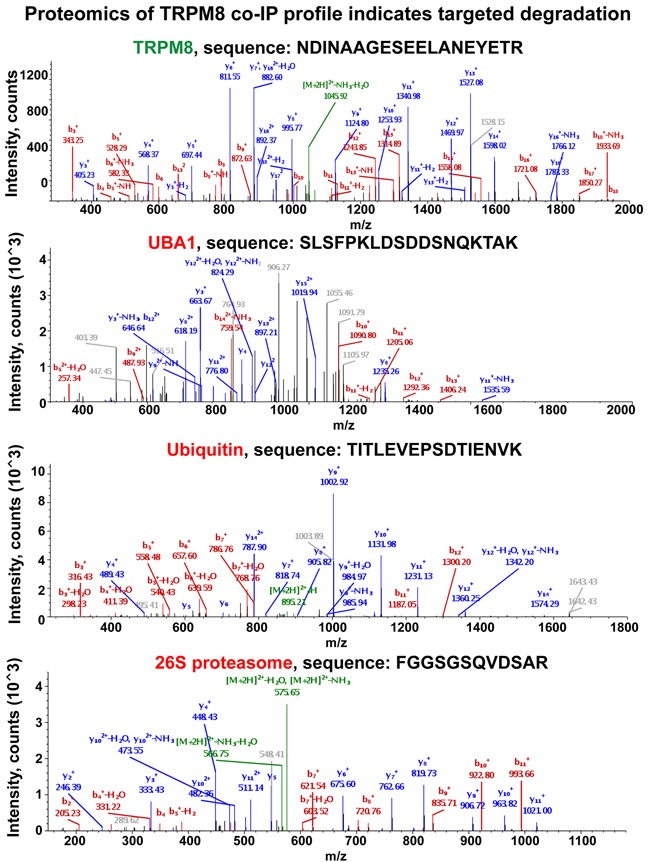
Proteomic analysis of TRPM8 and its interacting partners IP-ed from the LNCaP control cells indicates the presence of the proteins implicated in its targeted degradation Along with TRPM8 identification, the LC-MS/MS protein identity analysis resulted in obtaining multiple specific peptides corresponding to UBA1, ubiquitin, and 26S proteasome. The representative spectra corresponding to these proteins are shown in the figure. y- and b- peptide fragments ions are labeled in blue and red, respectively. More representative spectra are shown in Supplementary Figure 4.

Screening all the immunoprecipitated proteins using mass spectrometry enabled a tool to construct a comprehensive global view on TRPM8 turnover in the prostate cancer cells. Intriguingly, we found that the majority of the proteins co-precipitated were those that are implicated in degradation pathways. Figure 5 illustrates a few representative spectra of proteins detected at high abundance levels, which include TRPM8, UBA1, ubiquitin, as well as 26S proteasome obtained from control LNCaP cells.

Mass spectrometry (MS) based proteomics is considered as one of the most advanced techniques for validating protein identity. When MS is used in conjunction with immunoprecipitation techniques, it can provide the basis for establishing specific protein-protein interactions. The greatest challenge in this process is that sometimes it can result in overlapping molecular masses of the peptides and thus in hitting unspecific targets. To maximize the scrutiny for identification of specific TRPM8-interacting partners, we have not only limited the analysis to the interpretation based upon the SequestHT database search results, but also rigorously inspected the product ion spectra of the resulting peptides. To precisely validate the specificity of the identified proteins we demonstrate the representative spectra of the original peptide hits derived from the MS/MS. The pool of hits that resulted in specific spectra and, thus, the identification, were interpreted as TRPM8-interacting partners. In contrast, protein hits that did not meet this criterion were excluded from the list of potential TRPM8-interacting partners. Although the analysis resulted in revealing multiple interacting proteins, to maintain a particular focus on the biogenesis of TRPM8 here we report only the targets implicated in proteolysis. The representative spectra of the identified proteins are shown in Figure 5, and Supplementary Figures 3A-3H and 4A-4I. Furthermore, the spectra of specific targets that derived from various bands on the gel are displayed in the Supplementary Figure 4A-4I.

**Figure 6:**
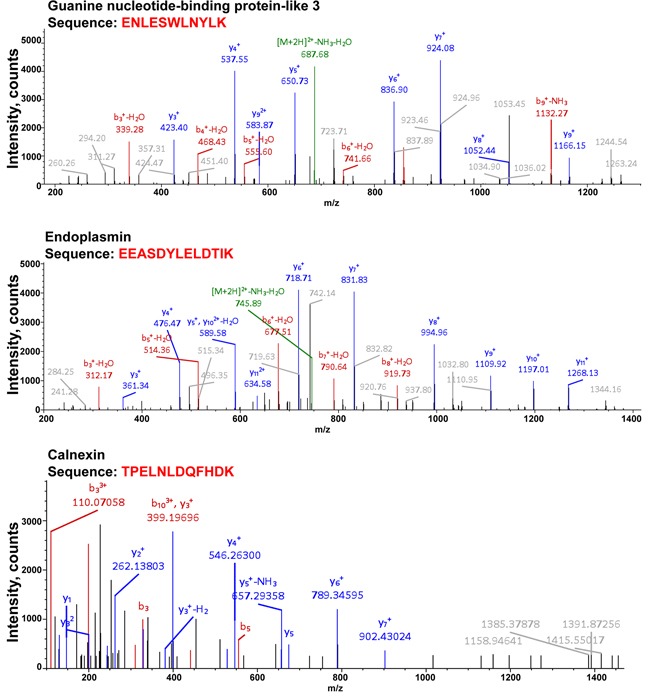
Proteomic analysis of TRPM8 and its interacting partners after the drug treatment indicates the presence of the proteins implicated in the ER quality control or stabilizing function Along with TRPM8 identification, the LC-MS/MS protein identity analysis resulted in obtaining multiple specific peptides corresponding to guanine nucleotide-binding protein-like 3 (GNL3), endoplasmin, and calnexin, detected at high abundance levels. The representative spectra corresponding to these proteins are presented in the figure. y- and b- peptide fragments ions are labeled in blue and red, respectively.

**Figure 7:**
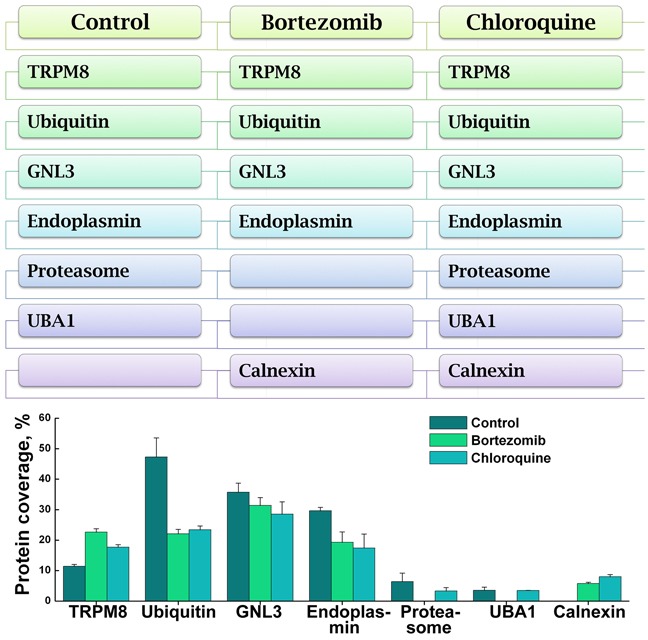
Distribution of TRPM8 and its interacting partners in the control and proteolysis inhibitor-treated LNCaP cells The scheme (top panel) summarizes the protein profiles related to TRPM8 and its interacting partners detected in the MS proteome analysis. The protein coverage and distribution in the control and drug-treated samples are shown in the graph (bottom panel). The categories include the control-untreated LNCaP cells, bortezomib-, and chloroquine-treated LNCaP cells. The conditions of the drug treatment are the same as described in the legend to Figure 4.

The MS analysis also indicated the presence of other regulatory proteins, including those implicated in the regulation of ER-associated degradation and quality control, endoplasmin and calnexin, as well as guanine nucleotide-binding protein-like 3 (Figure 6). Endoplasmin was detected at high-abundance levels during both the control and drug treatment conditions. Endoplasmin, also known as the heat-shock protein 90 (HSP90), is implicated in the ER-associated degradation and protein quality control [11, 12]. Calnexin was obtained in the proteomic analysis of the bortezomib- and chloroquine-treated cells. Calnexin is thought to play a role in the quality control apparatus of the ER by the retention of unfolded proteins [13]. It is plausible, that both endoplasmin and calnexin are in control of TRPM8 clearance from the ER, and may indicate another level of regulation and control. In contrast, the guanine nucleotide-binding protein-like 3 (GNL3) could play a role to prevent TRPM8 degradation. GNL3 is known for its ubiquitin-preventive actions [14, 15], and thus could play a role in stabilizing the TRPM8 protein, and it was found under all experimental conditions. The TRPM8-interacting partners implicated in the channel turnover in prostate cancer cells are shown in Figure 7.

**Figure 8:**
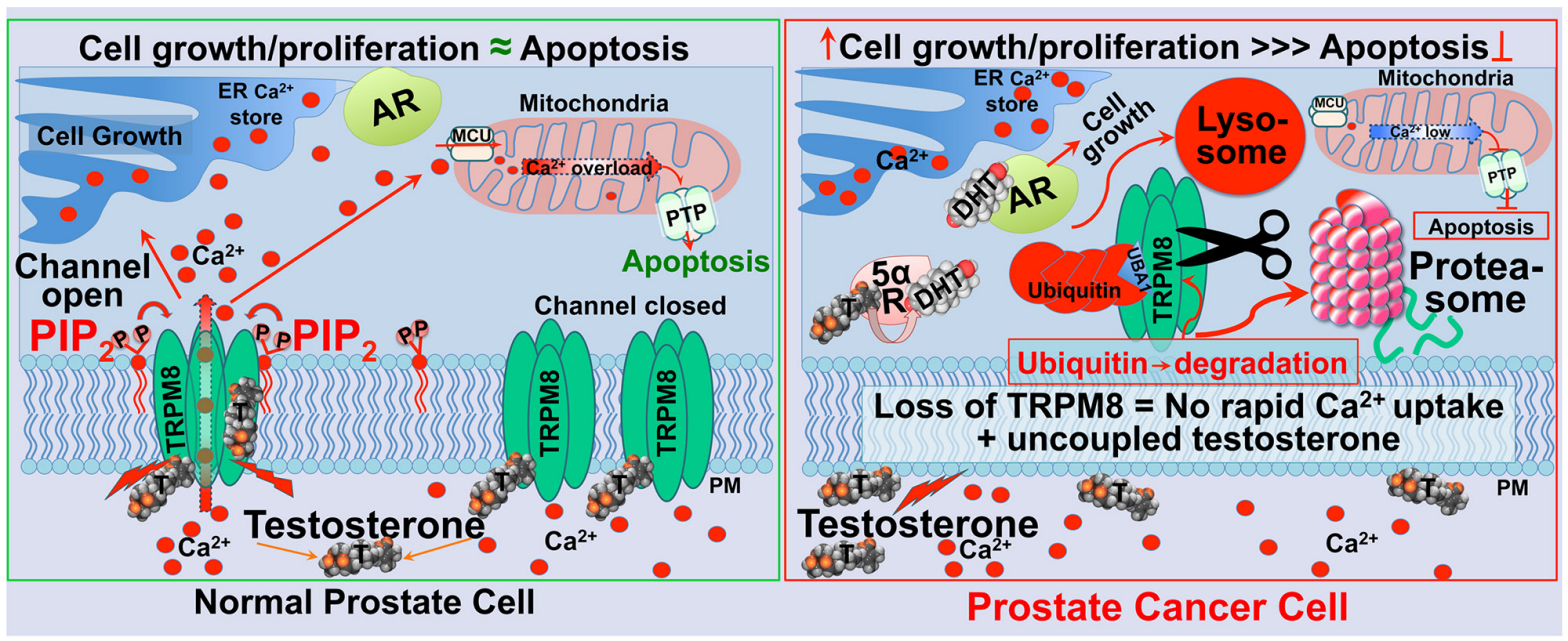
Schematic representation of TRPM8 as an ionotropic testosterone receptor in the normal prostate, regulating Ca^2+^ homeostasis (left panel), and enhanced TRPM8 degradation *via* lysosomal and proteasomal pathways in the prostate cancer (right panel) Testosterone-induced TRPM8 activation leads to rapid Ca^2+^ uptake, thus causing an increase in cytosolic Ca^2+^ concentration. This process engages TRPM8 and testosterone in the regulation of Ca^2+^ homeostasis in normal prostate. The activation of TRPM8 by testosterone is maintained by the acute desensitization mechanism most likely *via* depletion of the prime TRPM8 co-factor PIP_2_. Balanced Ca^2+^ homeostasis controls the rates of cell growth, proliferation, and apoptosis. Elevated cytosolic Ca^2+^ might result in excessive mitochondrial Ca^2+^ uptake through the Mitochondrial Calcium Uniporter (MCU), which may induce activation of the Permeability Transition Pore (PTP), swelling of the mitochondria, rupture of the outer membrane, and release of cytochrome-C and other pro-apoptotic molecules. TRPM8 channel desensitization will balance its testosterone-induced potentiation. During prostate cancer development TRPM8 is aggressively removed from the plasma membrane by targeted degradation *via* proteasomal and lysosomal pathways. This mechanism abolishes rapid Ca^2+^ uptake, thus reducing Ca^2+^-induced cytotoxicity and the rates of apoptosis. Simultaneously, the absence of TRPM8 on the plasma membrane could lead to uncoupled testosterone. Increased testosterone is converted to DHT by 5α-reductase, and activates AR that drives tumor progression.

Together, the results concluded here and in our previous work [8] suggest that testosterone-evoked TRPM8 activity plays a role in the regulation of Ca^2+^ homeostasis in the prostate epithelium [4, 5]. This regulatory role of TRPM8 is disrupted during prostate cancer development caused by the intense protein degradation that might lead to diminished rates of rapid Ca^2+^ intake, and simultaneously, uncoupled testosterone levels in the absence of its high-affinity receptor [5]. This proposition for the role of TRPM8 in prostate cancer is depicted in the scheme, presented in Figure 8.

## DISCUSSION

The presence of TRPM8 mRNA in prostate cancer at high expression levels attracted attention towards this channel as an opportunistic prognostic marker [6]. However, uncertainty remained regarding how well the up-regulated TRPM8 mRNA levels correlate with the protein expression levels and the channel localization. It is particularly interesting that TRPM8 expression pattern differed between the normal prostate and the cancer cells. Previously, Prevarskaya's group demonstrated a very distinctive pattern of TRPM8 expression on the plasma membrane of differentiated normal prostate tissues [7]. On the other hand, testing the TRPM8 expression profile and responsiveness to its agonists in LNCaP cells indicated the lack of the channel expression on the plasma membrane of prostate cancer cells [16]. Attempting to address this peculiar difference in TRPM8 expression pattern, we recently demonstrated that the TRPM8 protein is targeted for degradation in LNCaP cells *via* ubiquitination [8]. Further, inhibition of the initial ubiquitin-activating enzyme UBA1 along with AR led to a partial recovery of TRPM8 on the plasma membrane. The rescuing of functional TRPM8 channels, in turn, negatively impacted cancer cells growth and proliferation and induced apoptosis, which was evidenced by TUNEL staining and transmission electron microscopy [8]. These results suggested that TRPM8 plays a tumor suppressor role. However, the ultimate question remained whether TRPM8 degradation also takes place in prostate cancer patients, and if so can its recovery be used as a tool to control the tumor growth.

In the present work, we documented that the TRPM8 expression profile indeed undergoes substantial rearrangements during prostate cancer progression, and the protein localization is significantly altered. In healthy individuals, TRPM8 expression preferentially demonstrated plasma membrane and ER pattern, whereas the channel was severely internalized in the tumor tissues. These findings confirmed that TRPM8 is targeted for degradation in prostate cancer, and indicated the reliability of using the LNCaP cell model in the given paradigm.

We recently revealed that TRPM8 down regulation in LNCaP cells was accomplished by the ubiquitination, which was accessed using Western blot analysis indicating the presence of TRPM8 degradation products in IP probes. Furthermore, we showed that the proteolytic remnants are precipitated with the ubiquitin antibody-probed beads in ubiquitination assay, thus indicating that TRPM8 proteolysis is initially triggered through the ubiquitination cascade [8]. Biotinylation, live imaging, and Ca^2+^ imaging experiments further confirmed TRPM8 degradation and its recovery upon UBA1 and AR inhibition [8]. However, it was not clear whether ubiquitylated TRPM8 is then targeted to lysosomal or proteasomal proteolytic machinery, or perhaps both. Generally, ubiquitins can target proteins for degradation *via* both pathways [17]. It is possible that cancer cells utilize additional proteolytic tools to control specific targets. Therefore, to determine the steps of TRPM8 proteolysis in PC we screened both pathways. For this purpose a pharmacological approach was utilized, using inhibitors for lysosomal and proteasomal machinery with their consequent effect on TRPM8 recovery.

### Elements of TRPM8 degradation machinery in prostate cancer

A high-throughput proteome analysis in the present study uncovered the molecular mechanism of TRPM8 biogenesis in the prostate cancer cells. Mass spectrometry evaluation of TRPM8-interacting partners one by one revealed the involvement of the key components of the proteolytic machinery specifically utilizing the TRPM8 protein. Among the critical regulators of this mechanism the ubiquitin cascade proteins, including UBA1, ubiquitin, polyubiquitin, as well as 26S proteasome appeared at high abundance. Furthermore, the LC-MS/MS experiments indicated the presence of GlyGly-motif on the lysine residue (Lys) of ubiquitin (GlyGly-Lys-motif) in the untreated LNCaP cells. Importantly, this GlyGly-motif was present on Lys48 residue, which is a poly-ubiquitination site with the particular functional characteristics.

The functional regulation of polyubiquitin chains is determined depending upon the Lys residue of the ubiquitin that is linked. For instance, the Lys6-linkage of ubiquitins can be implicated in DNA replication and repair [18]; Lys11-linked chains are involved in endoplasmic reticulum-associated degradation (ERAD) and cell cycle regulation [19]; Lys29-linked chains target proteins for lysosomal [20] and proteasomal degradation [21]. Polyubiquitin with Lys63-linkage regulates various cellular processes including DNA repair [22, 23], signal transduction [24], protein trafficking [25], and ribosomal protein synthesis [26]. Polyubiquitin with Lys48-linkage is the most prominent for the proteasomal degradation pathway [9]. In this regard, co-precipitation of Lys48 polyubiquitin with TRPM8 detected in the mass spectrometric analysis is, therefore, a strong indication that TRPM8 undergoes degradation through the proteasomal pathway. Indeed, the presence of 26S proteasome has also been repeatedly obtained among the TRPM8 interacting partners detected in LC-MS/MS.

Pharmacological inhibition of proteasomal degradation with bortezomib resulted in the recovery of full-length TRPM8. However, TRPM8 recovery was also profound after the treatment with the lysosomal inhibitor chloroquine. This scheme indicates that TRPM8 hydrolysis in the prostate cancer cells is maintained *via* both proteolytic mechanisms.

Considering that the TRPM8 channel expression pattern in the normal prostate tissues is predominantly localized to the plasma membrane, its enhanced degradation in the cancerous tissues suggests rather a targeted elimination. However, what is the physiological role of TRPM8 channels in the context of the prostate epithelium, and why is the protein so avidly degraded in cancer cells? One of the plausible scenarios for the enhanced TRPM8 proteolysis could be a way for cancer cells to overcome consequences from the rapid Ca^2+^-uptake. We recently discovered that TRPM8 is a highly potent ionotropic testosterone receptor. Testosterone directly activated the channel (EC_50_ ~65 pM) and triggered a rapid testosterone-signaling mechanism [4, 5]. Testosterone-dependent TRPM8 activity, thus, is an effective tool to regulate Ca^2+^ homeostasis and maintain the cell cycle (Figure 8). Indeed, in another work from our lab, we demonstrated that recovery of TRPM8 on the plasma membrane induces apoptosis of LNCaP cells, which was significantly reduced with TRPM8 knock down [8]. These findings affirmatively indicate that testosterone-induced TRPM8 controls cell growth and proliferation and regulates the rates of apoptosis. How then is this mechanism maintained in the normal prostate epithelium?

In general, regulation of receptors activation and inactivation is tightly managed under the normal physiologic conditions. One of the critical elements of this regulatory paradigm is a tuned switch between the sensitization and desensitization modes of an ion channel. For many TRP channels, sensitization is evoked in an agonist-dependent manner. As for desensitization, it may occur at various molecular checkpoints. For instance, the phenomenon of desensitization has been well studied on the example of the heat and capsaicin receptor TRPV1. The acute desensitization of TRPV1 is markedly Ca^2+^ dependent and implicates various signaling pathways [27]. TRPV1 dephosphorylation by the phosphatase calcineurin is one of the mechanisms that underlines the channel's desensitization [28, 29]. Other mechanisms have also been described, including rapid depletion of phosphoinositide-4,5-diphosphate (PIP_2_), required for the channel function, where PIP_2_-store depletion was suggested to depend upon Ca^2+^-mediated PLC activation [30–32]. Noteworthy, the acute TRPM8 desensitization is also strictly PIP_2_-dependent [33].

The long-term desensitization of TRPV1 has also been well elucidated, and it radically differs from the acute mechanism. Sanz-Salvador et al. demonstrated that prolonged treatment of the cells with TRPV1 agonist capsaicin not only inhibited the channel activity, but also triggered its lysosome-mediated degradation [34]. This phenomenon in the long-term inactivation of the profound pain receptor, like TRPV1, is thought to be a critical adaptation mechanism, which is essential in avoidance of continuous pain perception during acute or chronic inflammatory conditions.

Taking into consideration an existence of these regulatory mechanisms for TRPM8, it is possible that similar short- and long-term desensitization could play regulatory roles in maintaining its activity in the prostate epithelium. In fact, TRPM8 activity requires the presence of PIP_2_, and depletion of PIP_2_ stores inhibits the channel [33]. Thus, loss of channel activity triggered by PIP_2_-depletion could be one of the acute desensitization mechanisms. The long-term channel desensitization has not been previously described for TRPM8, and it is possible that its enhanced degradation in the prostate cancer results from certain pathological changes in the regulatory adaptation mechanism followed by the sustained channel inactivation. The molecular levers of this mechanism are not well understood, but they could depend on the availability of the endogenous TRPM8 agonist testosterone [4, 5].

The relationship with the other androgen responsive proteins like androgen receptor (AR) could also be essential for regulating TRPM8 activity. It is important to note that androgen-TRPM8 and androgen-AR have opposing mechanisms of action, e.g. non-genomic versus genomic. Furthermore, they have reverse affinities for binding androgens. DHT has nearly 10-fold higher AR affinity than testosterone (i.e. K_d_ of ~100 pM for DHT versus ~1 nM for testosterone) [35], while testosterone has almost 1000-fold higher TRPM8 affinity than DHT (i.e. EC_50_ of ~65 pM for testosterone versus ~21 nM for DHT) [5]. Comparing both the receptors, thus, it appears that TRPM8 has higher sensitivity to testosterone than AR. Besides, TRPM8 presence on the plasma membrane initiates direct contact with the steroid, even before it enters the cell for further conversion to DHT. Thus, the TRPM8 protein appears to be the first immediate receptor for testosterone with all its subsequent physiological implications. Considering the margins of androgen availability and its distribution to its specific receptors would lead to two distinct mechanisms of action. Thus, in the case of AR, androgens in the form of DHT stimulate protein synthesis, cell growth, and proliferation; as in the case of TRPM8 androgens in the form of testosterone control cell cycle by maintaining balanced Ca^2+^ homeostasis (Figure 8). In this light, testosterone-TRPM8 actions present a tumor suppression mechanism. Thus, ensuring a sustained channel expression on the plasma membrane can lead to a novel therapeutic strategy in an anti-tumor defense mechanism.

## MATERIAL AND METHODS

### Cell culture

The prostate cancer cell line LNCaP was obtained from the American Type Culture Collection (Manassas, VA) and cultured as directed. Briefly, LNCaP cells were cultured in RPMI medium supplemented with 10% fetal bovine serum (Invitrogen) and 1% penicillin/streptomycin (Invitrogen). Human embryonic kidney HEK-293 cells were maintained in minimal essential medium (MEM) solution (Invitrogen, San Diego, CA) supplemented with 10% fetal bovine serum (Invitrogen) and 1% penicillin/streptomycin.

The cells were maintained in a 37 °C incubator in a 5% CO_2_ humidified atmosphere. The cells were transfected with the rat TRPM8 cDNA using the Effectene reagent (Qiagen, Chatsworth, CA). The TRPM8 stable cell line was developed with TRPM8 tagged with myc on the N-terminus as previously described [36].

### Immunoblotting and immunoprecipitation assay

Western blot analysis was done using equal amounts of protein separated by SDS-PAGE, transferred onto nitrocellulose or PVDF membranes (Biorad, Hercules, CA), incubated with 1:1000 dilution of primary antibodies, and subsequently incubated with 1:1000 dilution of species-specific, HRP conjugated secondary antibody according to the standard protocol [37]. Immunoprecipitation assays were carried out by incubating about 1000 µg of cell lysates with the required specific primary antibody (2 µg) overnight at 4 °C on a rotating shaker. Around 70 μL of protein A/G agarose beads (Miltenyi Biotech, Auburn, CA) were added to the above complex and incubated on ice for 30 min. These beads were passed through MACS 20 µm separation columns (Miltenyi Biotech) and the bound immunoprecipitates were eluted according to the manufacturer's instructions. The immunoprecipitates were then separated on the SDS-PAGE, stained with Coomassie blue and processed for MS analysis, or immunoblotted using specific primary antibodies.

### Immunohistochemistry

Prostate cancer tissue microarray (US Biomax, Rockville, MD), containing 60 cases of prostate adenocarcinoma (grade 1-4) and 9 cases of normal prostate tissues were processed for immunohistochemical analysis according to the standard protocol [38]. The pathology description on Gleason score and grade is provided by US Biomax. The tissue sections were deparaffinized in xylene and rehydrated in graded ethanol solutions. Antigen retrieval was carried out with 10 mM citrate buffer (pH 6) at boiling temperature for 60 min and permeabilization in 0.1% Triton-X-100. The sections were blocked using 10% BSA in 1X PBS and were incubated with primary rabbit TRPM8 antibody (Pheonix Pharmaceuticals, Burlingame, CA) overnight at 4°C, washed with PBS and incubated with fluorescent-labeled, species-specific secondary antibodies (Alexa Fluor) at 1:500 dilution for 1 h at room temperature. Before mounting, the slides were washed with PBS and incubated for 5 min with 4′-6-diamidino-2-phenylindole (DAPI) for nuclear staining and analyzed using confocal microscopy (Zeiss LSM 510 upright confocal microscope, Toronto, ON) at 40X magnification.

### Mass spectrometry analysis

After the immunoprecipitation, TRPM8 preps were separated by SDS-PAGE and stained with Coomassie blue. All the resulted bands were excised from the gel and digested with trypsin for mass spectrometry analysis according to Mann et al protocol [39], with some modifications. Briefly, the gel bands were reduced with 0.5 M dithiothreitol and alkylated with 0.7 M iodoacetamide. Gel bands were digested with trypsin (Promega, Madison, WI) for 12 h at 37°C. Peptides were extracted from the gel bands with 100 μL of a 50% acetonitrile 5% formic acid solution. The extract was dried by vacuum centrifugation (SPD SpeedVac Thermo Electron Corp. Waltham, MA); the tryptic peptides were resuspended in 20 μL of a 3% acetonitrile, 0.5% formic acid solution.

Liquid chromatography-tandem mass mass spectrometry (LC-MS/MS) was performed using a nano flow liquid chromatography system (Ultimate3000, ThermoScientific) interfaced to a hybrid ion trap-orbitrap high-resolution tandem mass spectrometer (VelosPro, ThermoScientific) operated in data-dependent acquisition (DDA) mode. Briefly, one microliter of each sample was injected onto a capillary column (4 μm Jupiter C18 manually packed on a 30 cm x 75um ID PicoFrit Column, New Objective) at a flow rate of 300 nl/min. Samples electro-sprayed at 1.2 kV using a dynamic nanospray ionization source. Chromatographic separation was carried out using 90 minute linear gradients (Mobile Phase A: 0.1% formic acid in MS-grade water, mobile phase B: 0.1% formic acid in MS-grade acetonitrile) from 3% B to 35% B over 60 minutes, then increasing to 95% B over 5 minutes. MS/MS spectra were acquired using both collision-induced dissociation (CID) and higher-energy collisional dissociation (HCD) for the top 15 peaks in the survey 30000-resolution MS scan. The .raw files were acquired (Xcalibur, ThermoFisher) and exported to Proteome Discoverer 2.0 (ThermoFisher) software for peptide and protein identification using SequestHT search algorithm (Full trypsin digestion with 2 maximum missed cleavages, 10 ppm precursor mass tolerance and 0.8 Da fragment mass tolerance). Database searching was done using the UniprotKB human database. Carbamidomethylcysteine was selected as static modification while oxidized methionine and phosporylations on S, T and Y were selected as dynamic modifications. Additionally, Gly-Gly dynamic modifications were selected on lysine residues to target ubiquitinated proteins.

### Statistical analysis

Statistical analysis was performed using Origin 9.0 software (Microcal Software Inc., Northampton, MA, USA). Statistical significance was calculated using one-way ANOVA followed by Fisher's LSD test and data were expressed as mean ± SEM. P <0.05 was considered to be significant. In all figures, statistical significance is labeled the following way: *P <0.05, **P <0.01, ***P <0.001, and ****P <0.0001.

## SUPPLEMENTARY MATERIALS FIGURES AND TABLES


