# Supplementary material for: High-throughput proteome analysis reveals targeted TRPM8 degradation in prostate cancer
Source: Oncotarget. 2016 Dec 26;8(8):12877–90. doi: 10.18632/oncotarget.14178 (PMC5355063; doi:10.18632/oncotarget.14178)
Supplement: Supplementary file 1 [file oncotarget-08-12877-s001.pdf]

High-throughput proteome analysis reveals targeted TRPM8 degradation in prostate cancer

SUPPLEMENTARY FIGURES

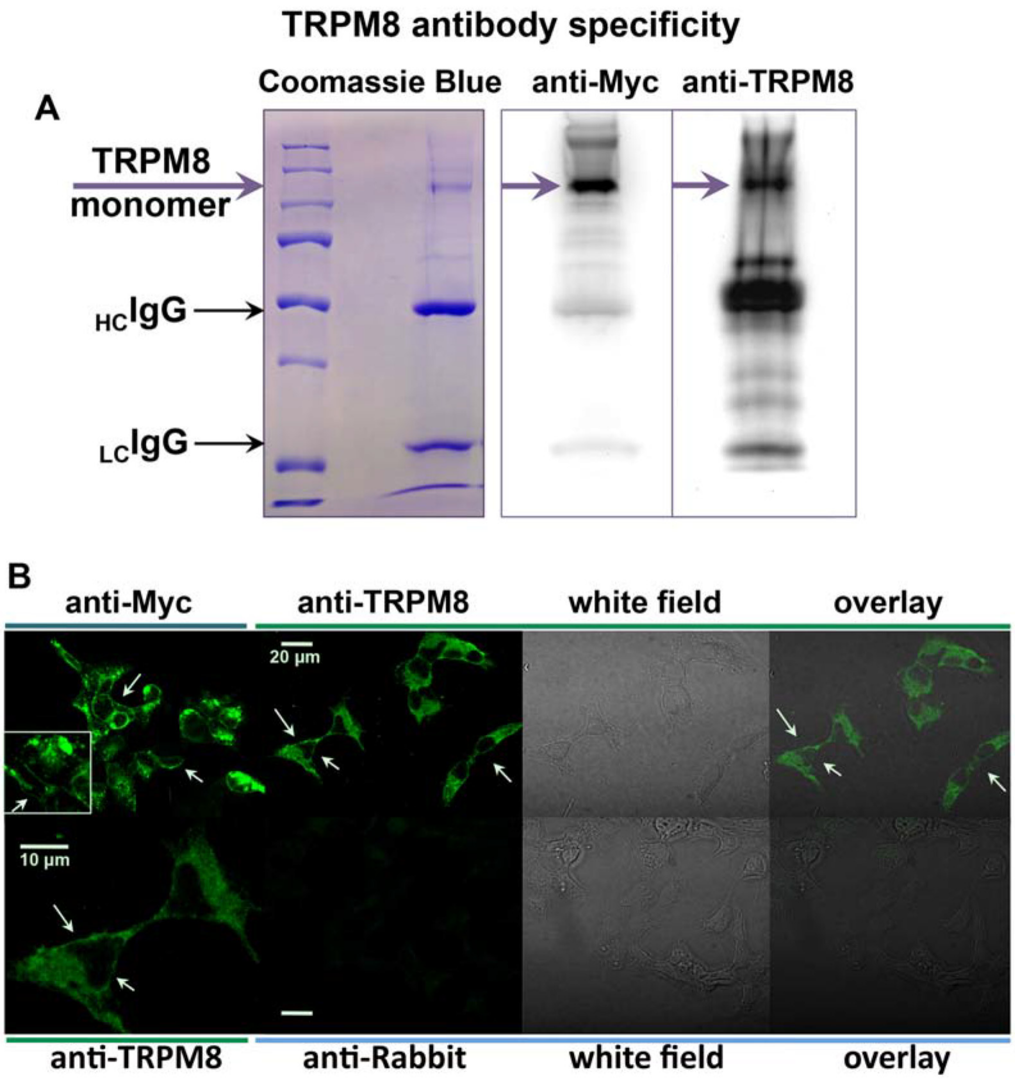

Supplementary Figure 1: Anti-TRPM8 antibody specificity indicated in Western Blot and immunocytochemistry, Related to Figure 1. A. Myc-tagged TRPM8 was IP-ed from the HEK-293 cells stably expressing the protein using anti-Myc-IgG conjugated to A/G magnetic beads. TRPM8 was eluted from the beads with SDS-loading buffer and separated on 10% SDS gel, where the homotetramer preferably migrates in the form of a monomer at ~130 kDa. TRPM8 was detected using Coomassie blue staining, and WB with anti-Myc-IgG and anti-TRPM8-IgG, where the profound monomer bands are indicated with the arrows. Heavy and Light Chain IgGs are indicated below (n = 3). **B.** Immunocytochemistry using both monoclonal anti-Myc or polyclonal anti-TRPM8 antibodies demonstrates typical TRPM8 distribution pattern localized to the PM and ER. Negative controls with using the secondary antibodies alone shows no staining (n = 6).

A

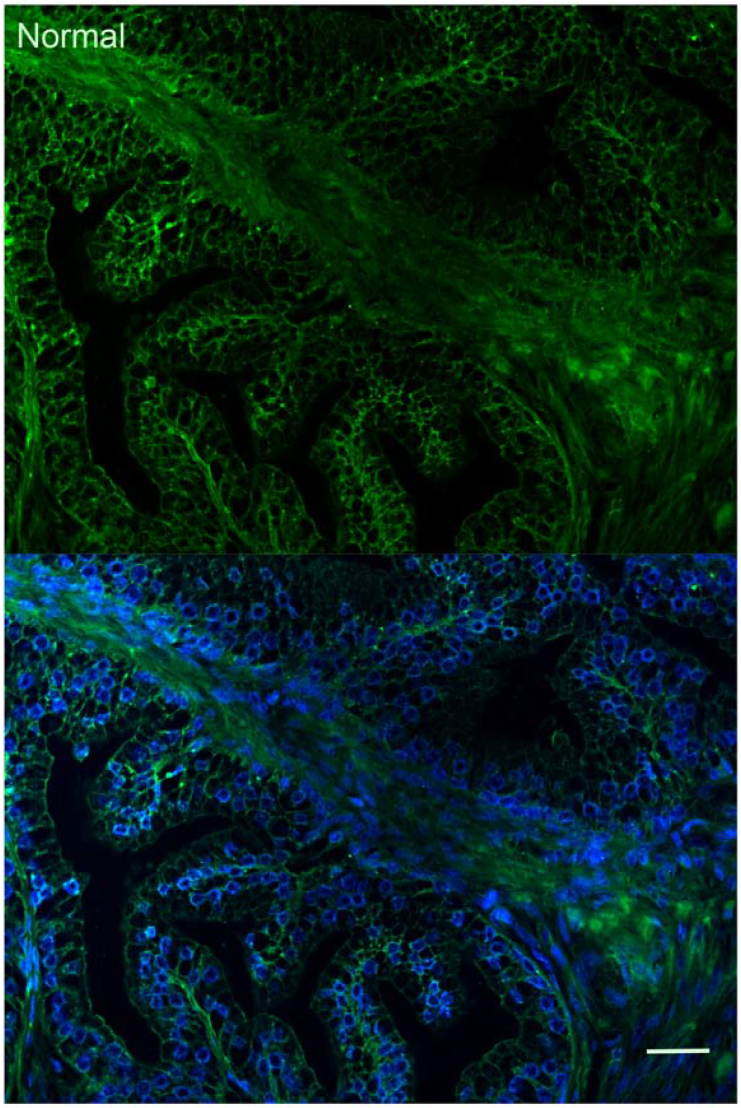

Supplementary Figure 2A: TRPM8 exhibits plasma membrane or ER pattern in the peripheral prostate epithelium of healthy individuals, Related to Figure 1. TRPM8 was detected with anti-TRPM8-IgG, and secondary AlexaFluor488. The nuclear staining visualized with DAPI. The bar scale is 20 µm. (*Continued*)

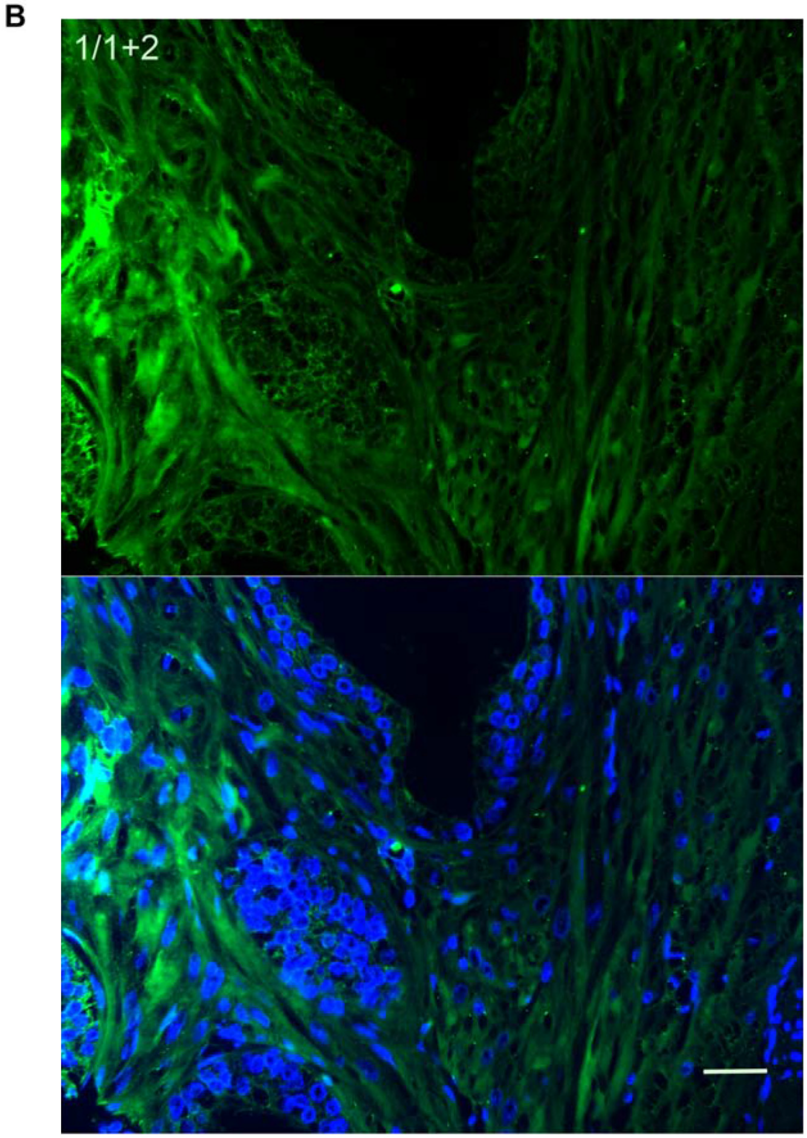

Supplementary Figure 2B: (*Continued*) TRPM8 expression pattern in the prostate cancer patient grade 1, Gleason score 1+2, Related to Figure 1. (*Continued*)

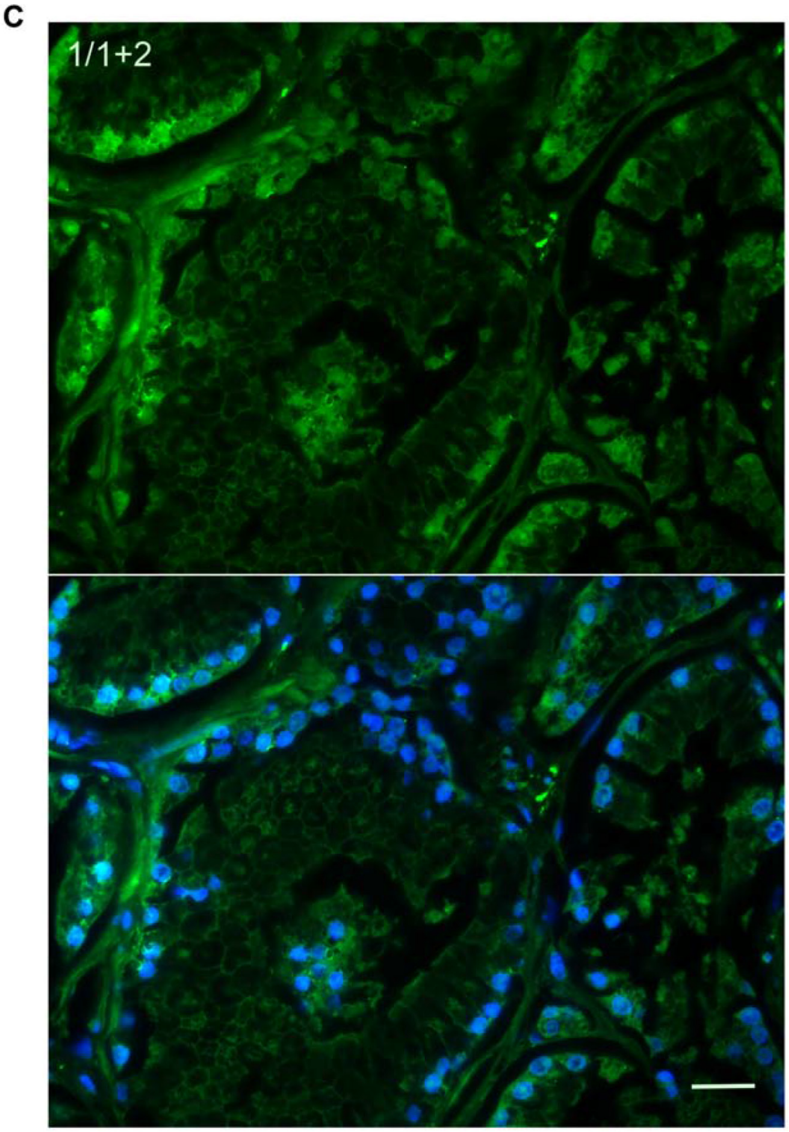

Supplementary Figure 2C: (*Continued*) TRPM8 expression pattern in the prostate cancer patient grade 1, Gleason score 1+2, Related to Figure 1. (*Continued*)

D

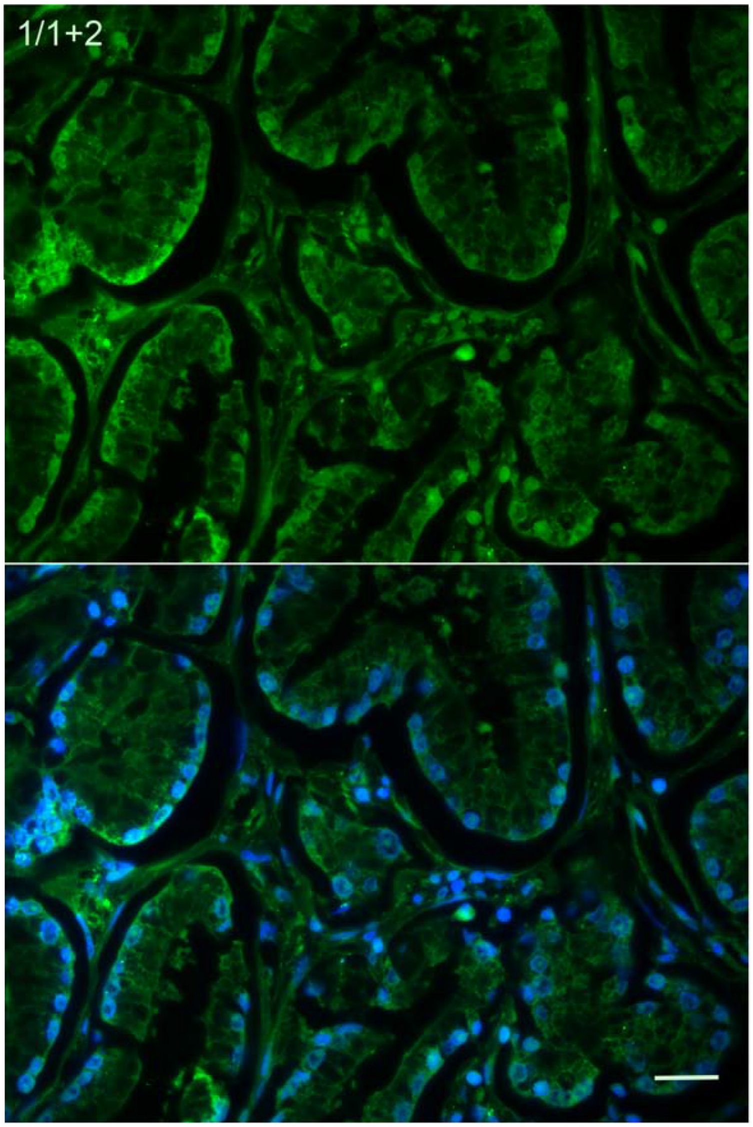

Supplementary Figure 2D: (*Continued*) TRPM8 expression pattern in the prostate cancer patient grade 1, Gleason score 1+2, Related to Figure 1. (*Continued*)

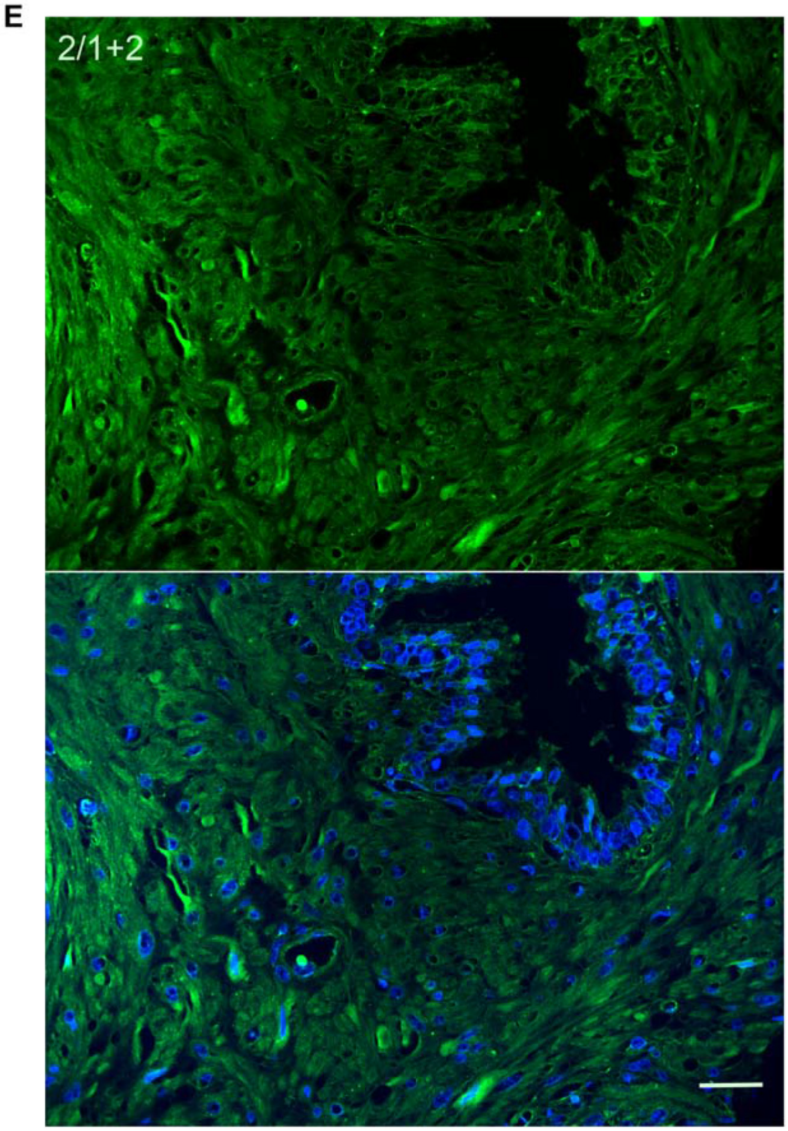

Supplementary Figure 2E: (*Continued*) TRPM8 expression pattern in the prostate cancer patient grade 2, Gleason score 1+2, Related to Figure 1. (*Continued*)

F

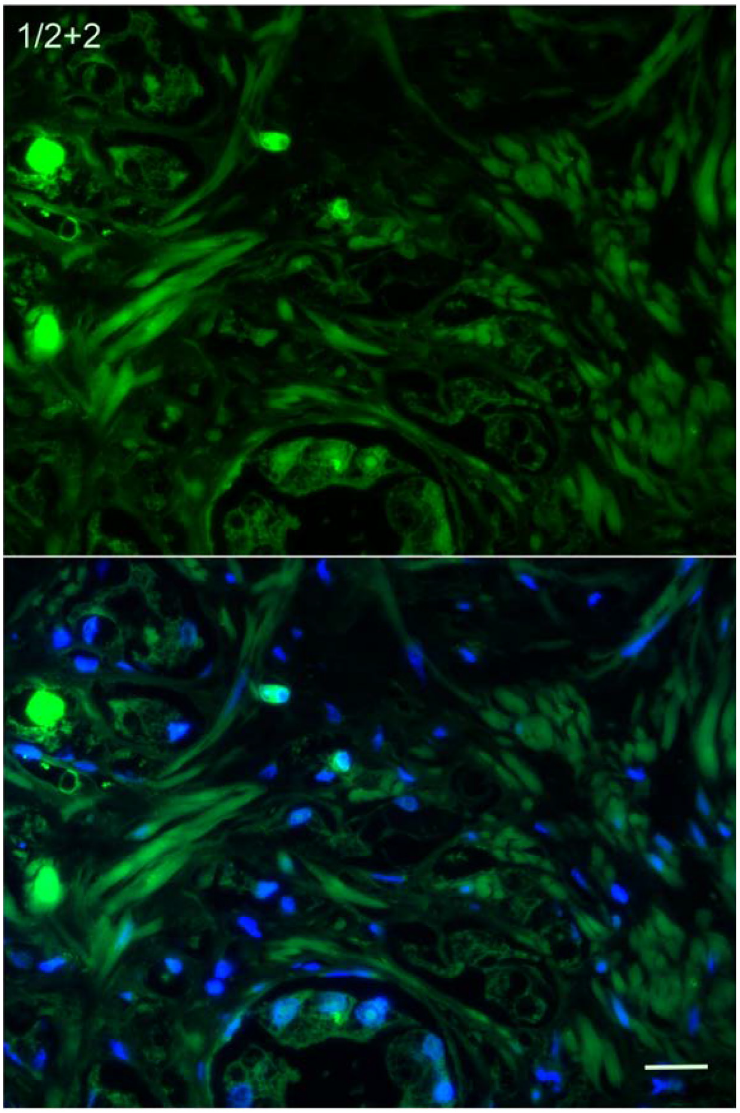

Supplementary Figure 2F: (*Continued*) TRPM8 expression pattern in the prostate cancer patient grade 1, Gleason score 2+2, Related to Figure 1. (*Continued*)

G

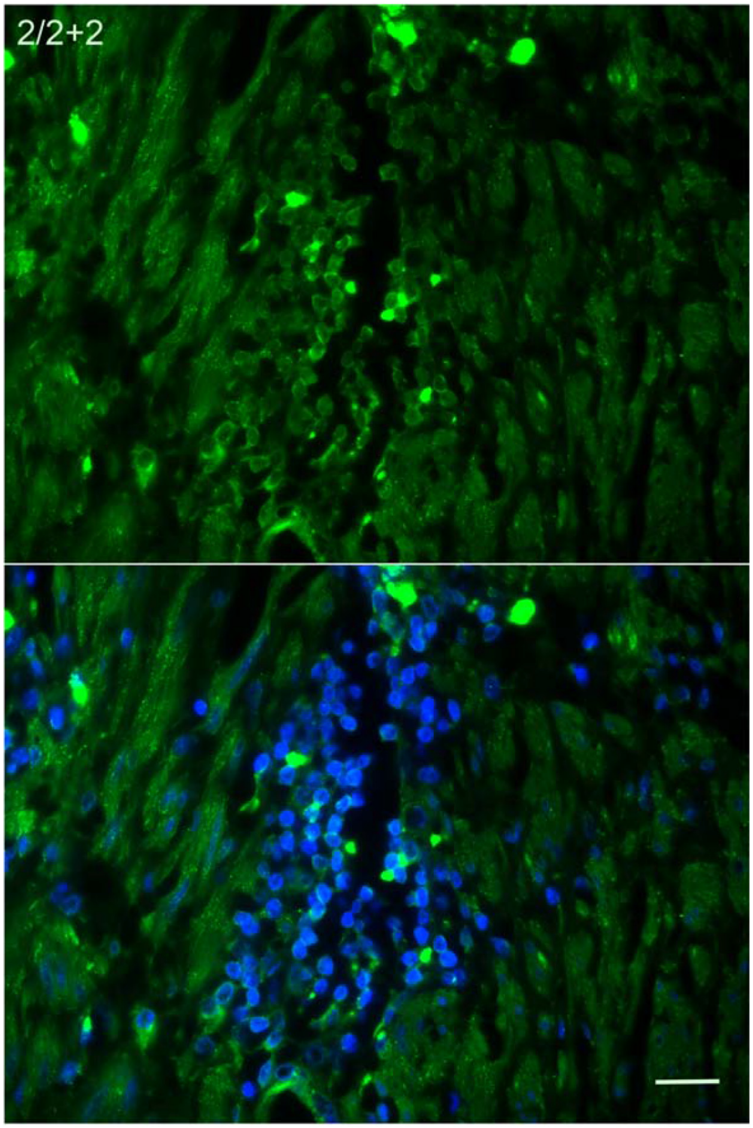

Supplementary Figure 2G: (*Continued*) TRPM8 expression pattern in the prostate cancer patient grade 2, Gleason score 2+2, Related to Figure 1. (*Continued*)

H

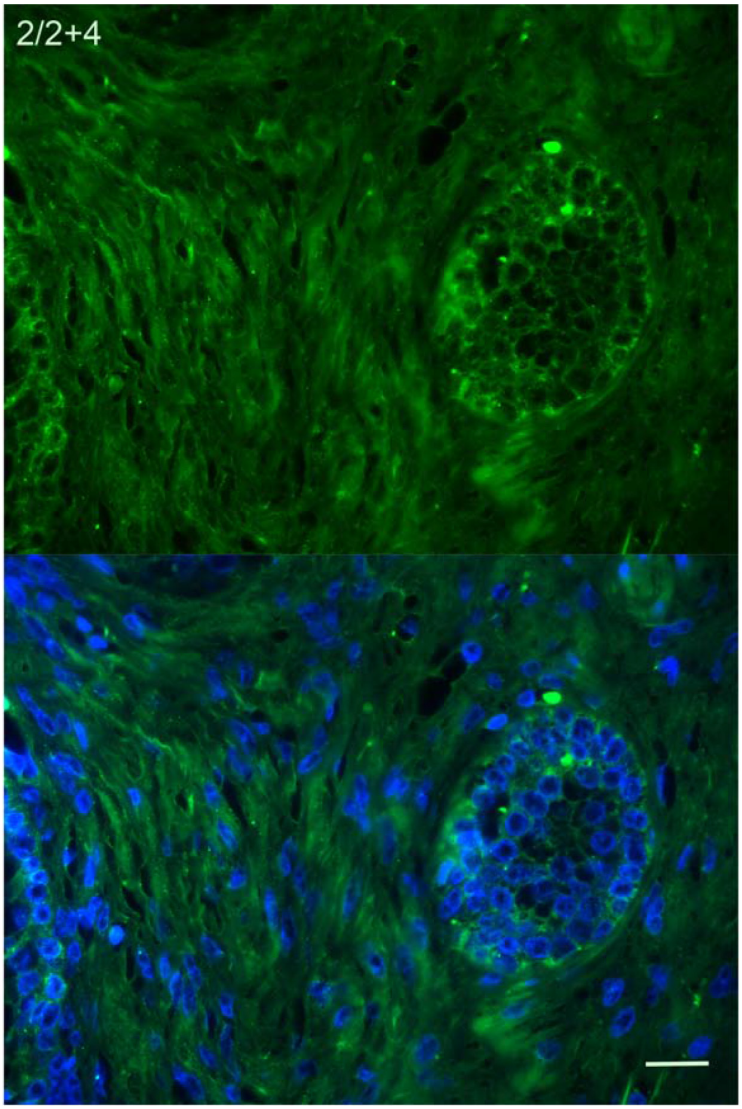

Supplementary Figure 2H: (*Continued*) TRPM8 expression pattern in the prostate cancer patient grade 2, Gleason score 2+4, Related to Figure 1. (*Continued*)

I

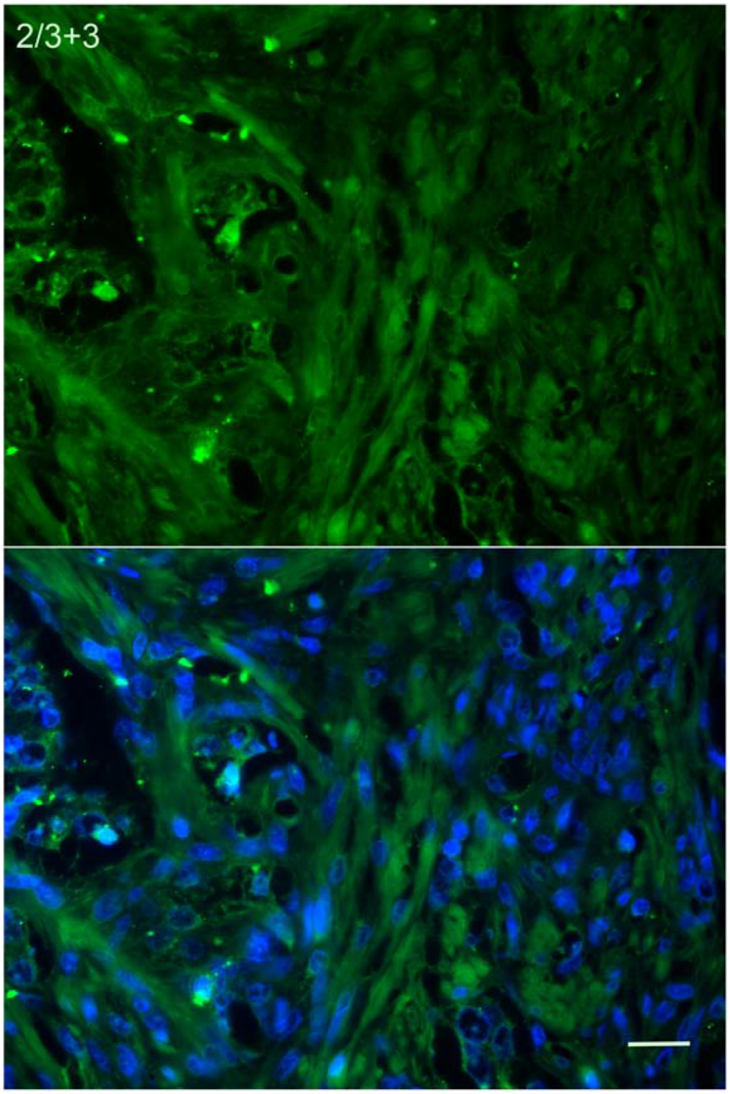

Supplementary Figure 2I: (*Continued*) TRPM8 expression pattern in the prostate cancer patient grade 2, Gleason score 3+3, Related to Figure 1. (*Continued*)

J

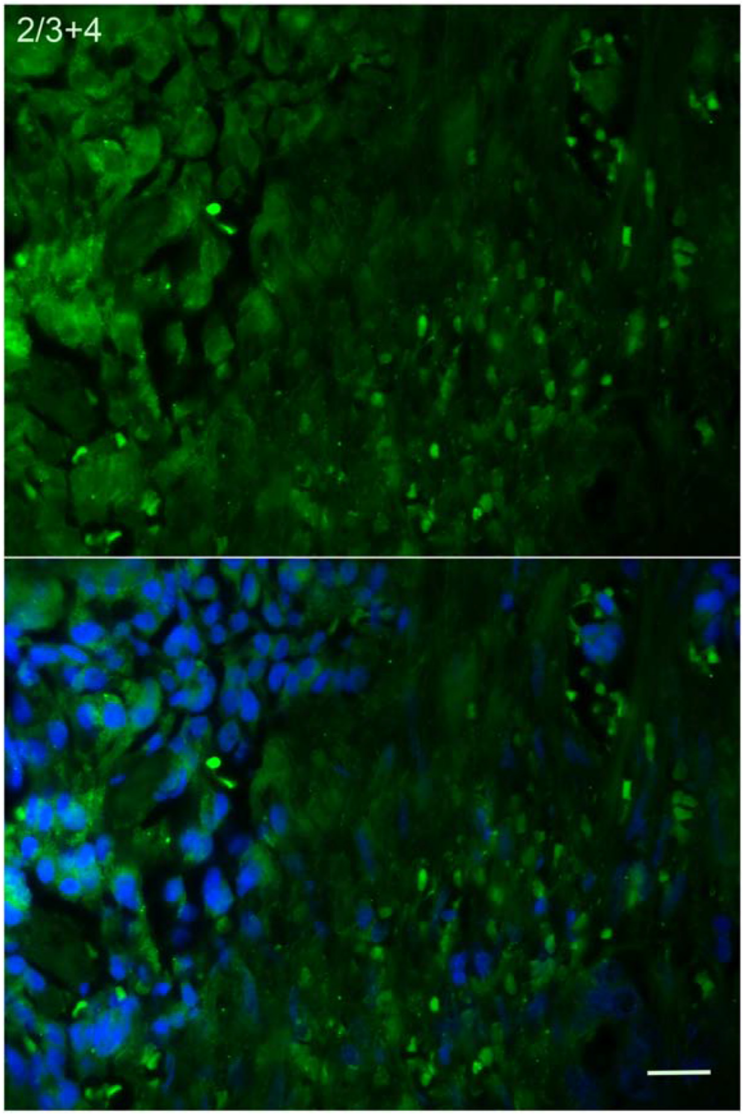

Supplementary Figure 2J: (*Continued*) TRPM8 expression pattern in the prostate cancer patient grade 2, Gleason score 3+4, Related to Figure 1. (*Continued*)

K

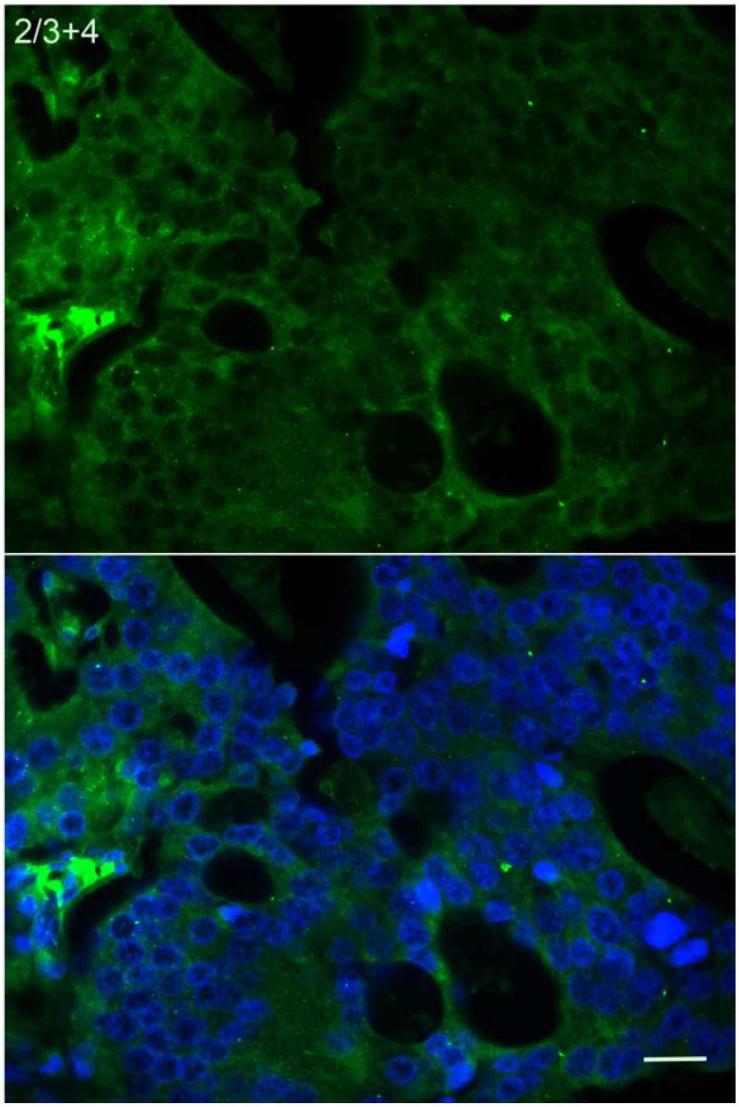

Supplementary Figure 2K: (*Continued*) TRPM8 expression pattern in the prostate cancer patient grade 2, Gleason score 3+4, Related to Figure 1. (*Continued*)

L

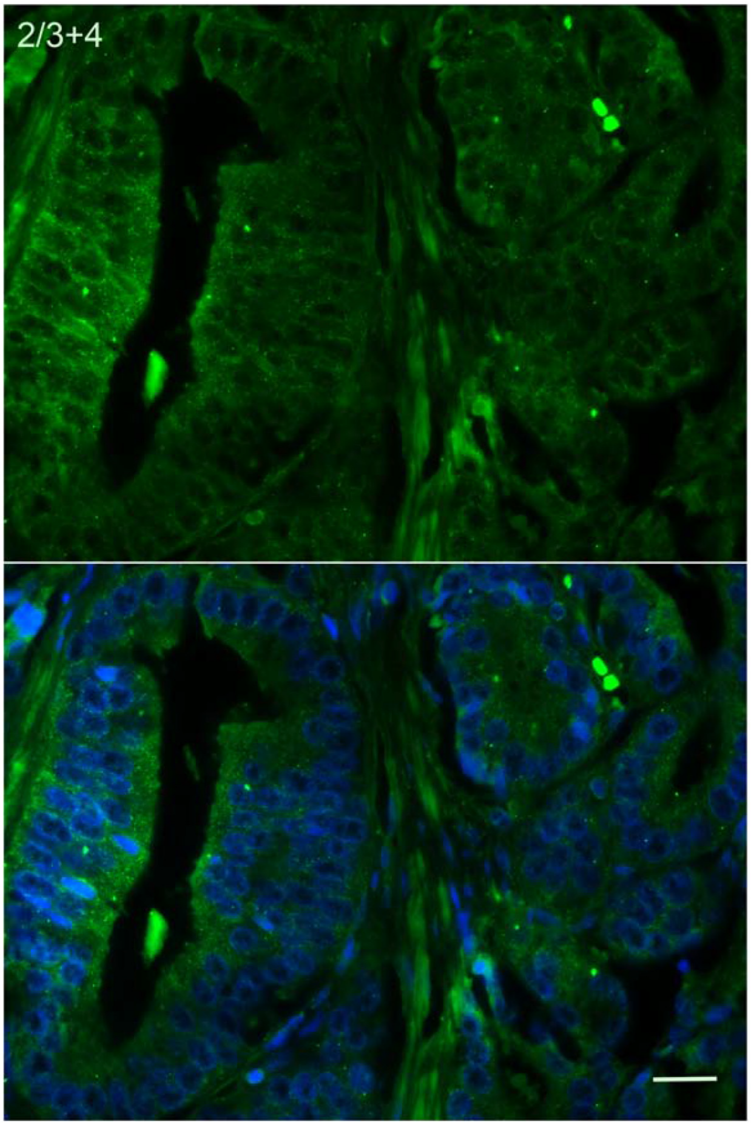

Supplementary Figure 2L: (*Continued*) TRPM8 expression pattern in the prostate cancer patient grade 2, Gleason score 3+4, Related to Figure 1. (*Continued*)

M

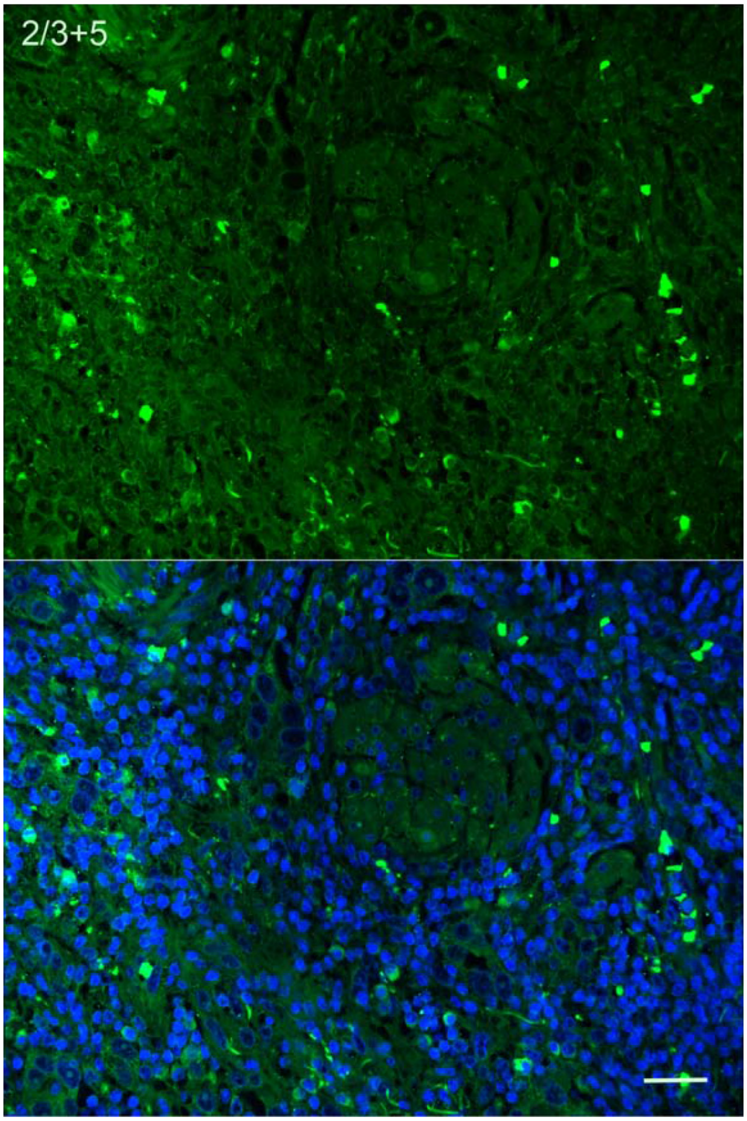

Supplementary Figure 2M: (*Continued*) TRPM8 expression pattern in the prostate cancer patient grade 2, Gleason score 3+5, Related to Figure 1. (*Continued*)

N

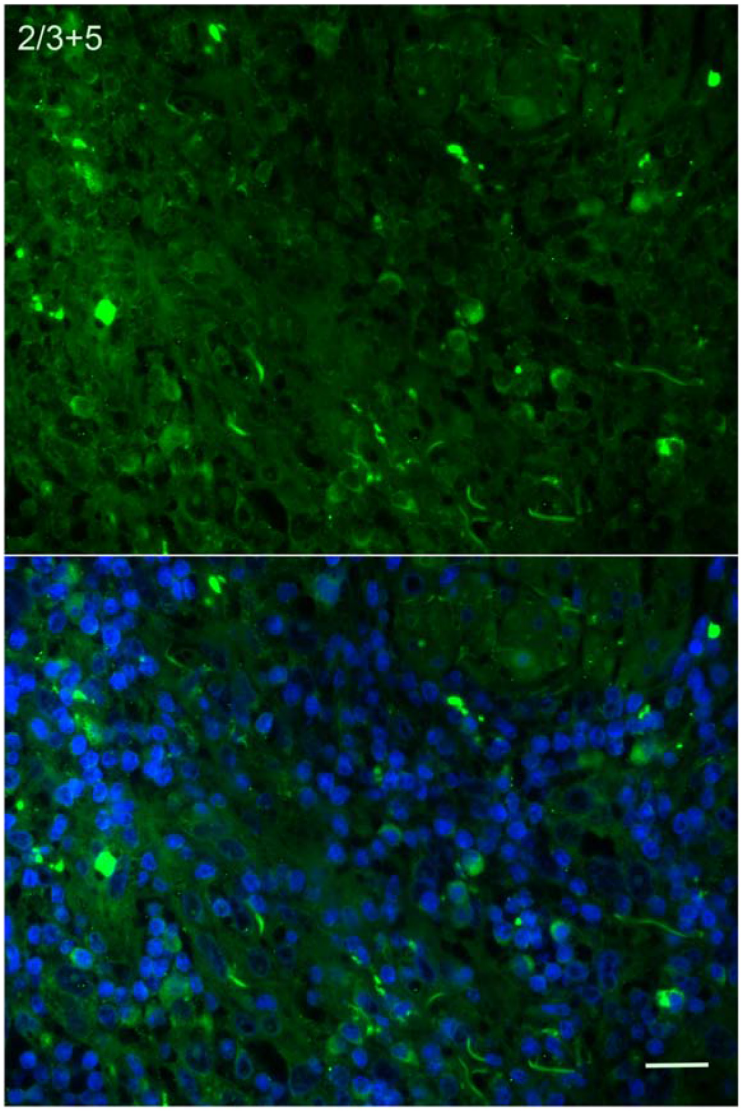

Supplementary Figure 2N: (*Continued*) TRPM8 expression pattern in the prostate cancer patient grade 2, Gleason score 3+5, Related to Figure 1. (*Continued*)

O

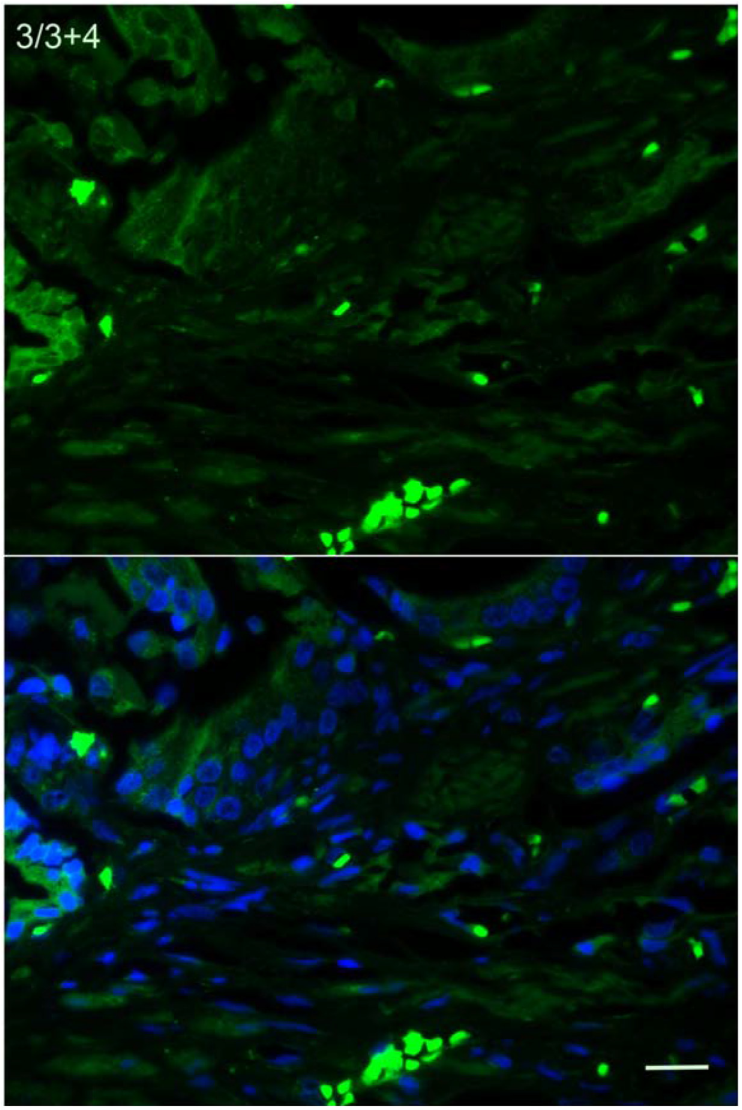

Supplementary Figure 2O: (*Continued*) TRPM8 expression pattern in the prostate cancer patient grade 3, Gleason score 3+4, Related to Figure 1. (*Continued*)

P

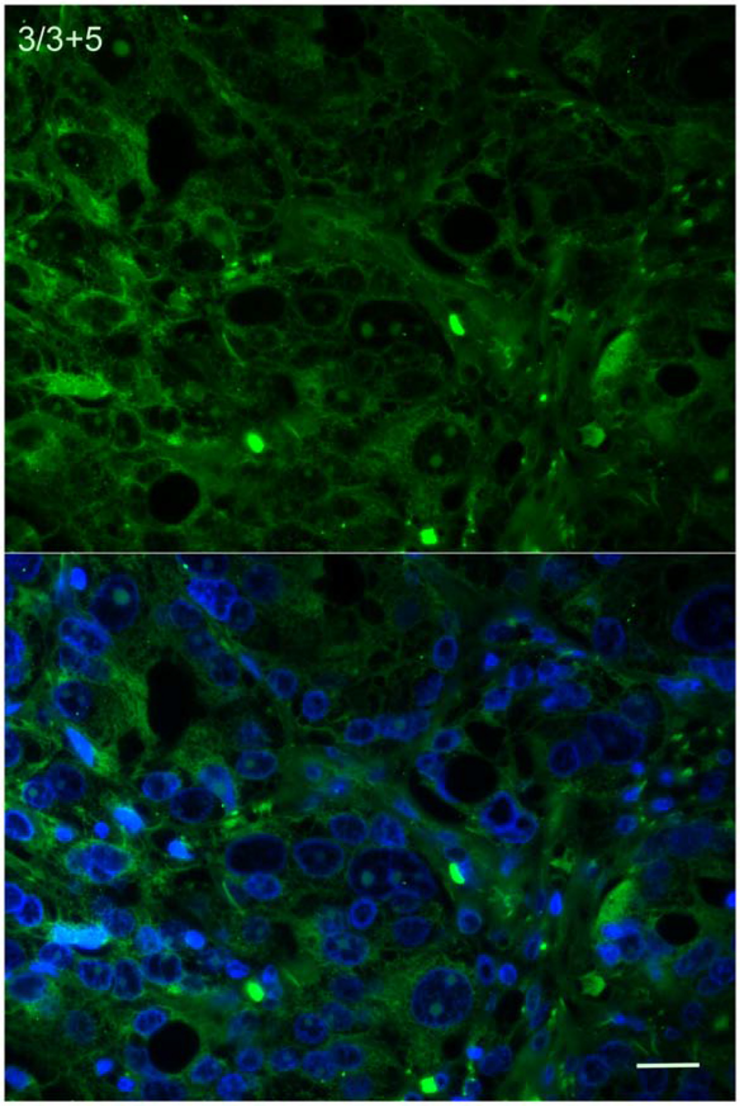

Supplementary Figure 2P: (*Continued*) TRPM8 expression pattern in the prostate cancer patient grade 3, Gleason score 3+5, Related to Figure 1. (*Continued*)

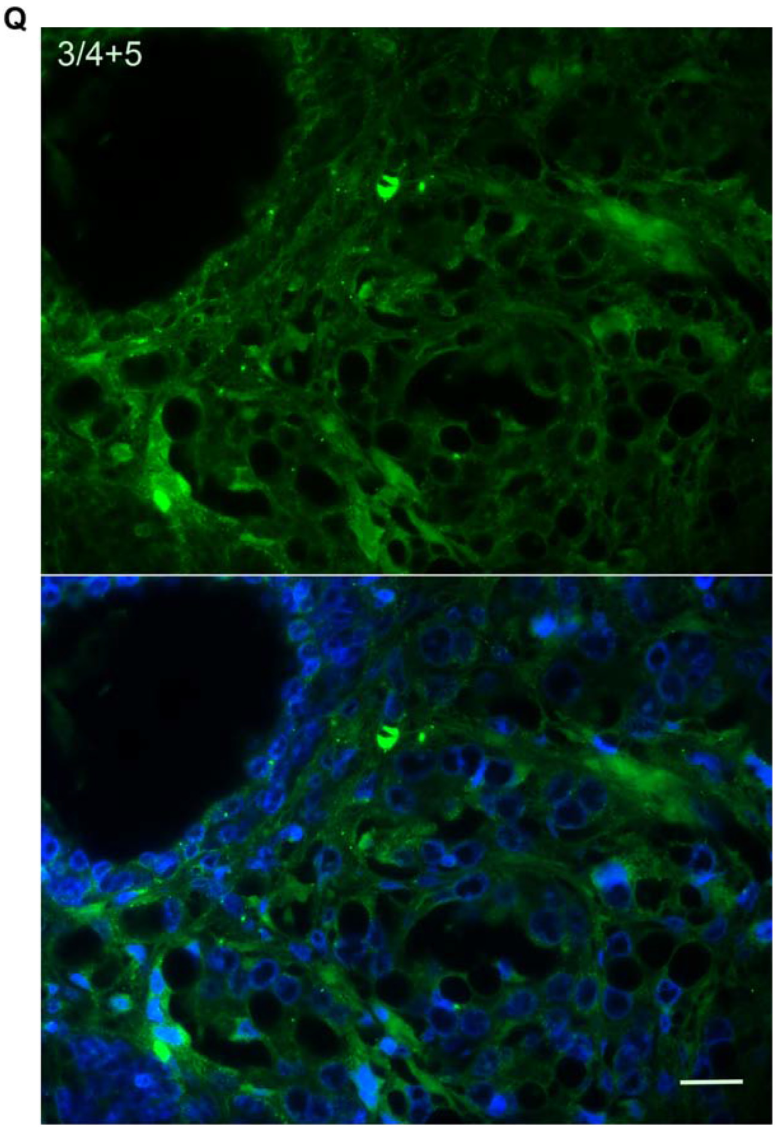

Supplementary Figure 2Q: (*Continued*) TRPM8 expression pattern in the prostate cancer patient grade 3, Gleason score 4+5, Related to Figure 1. (*Continued*)

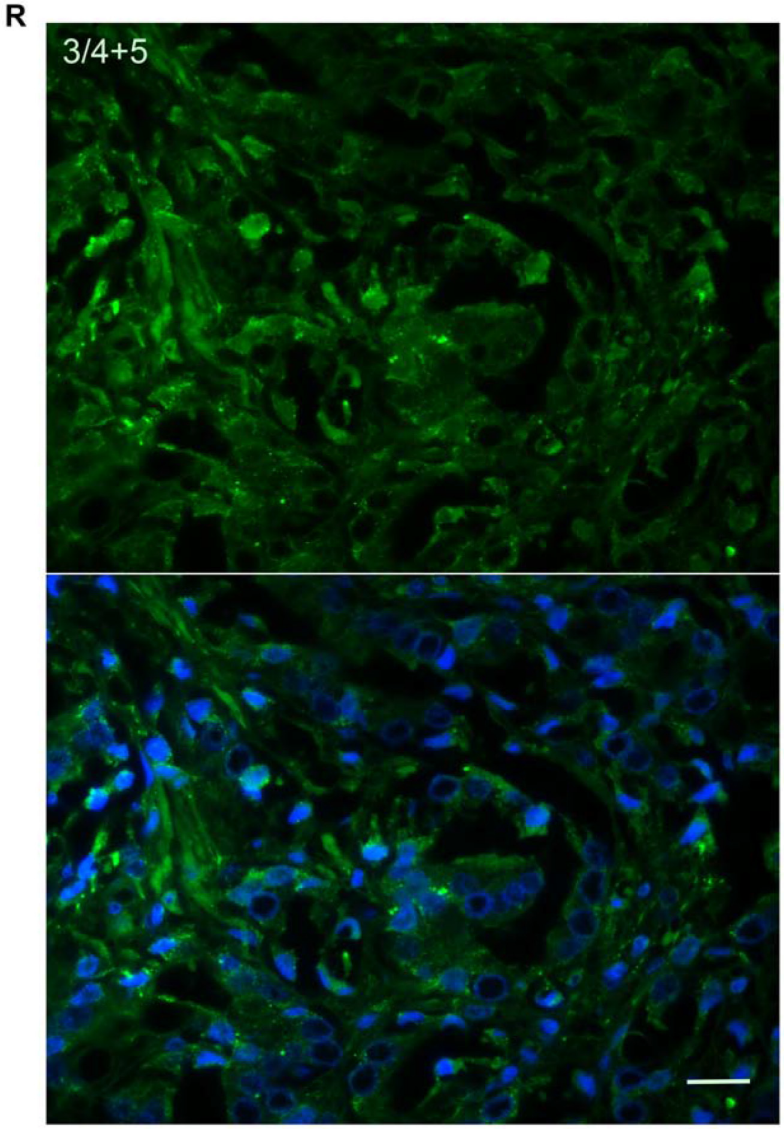

Supplementary Figure 2R: (*Continued*) TRPM8 expression pattern in the prostate cancer patient grade 3, Gleason score 4+5, Related to Figure 1. (*Continued*)

S

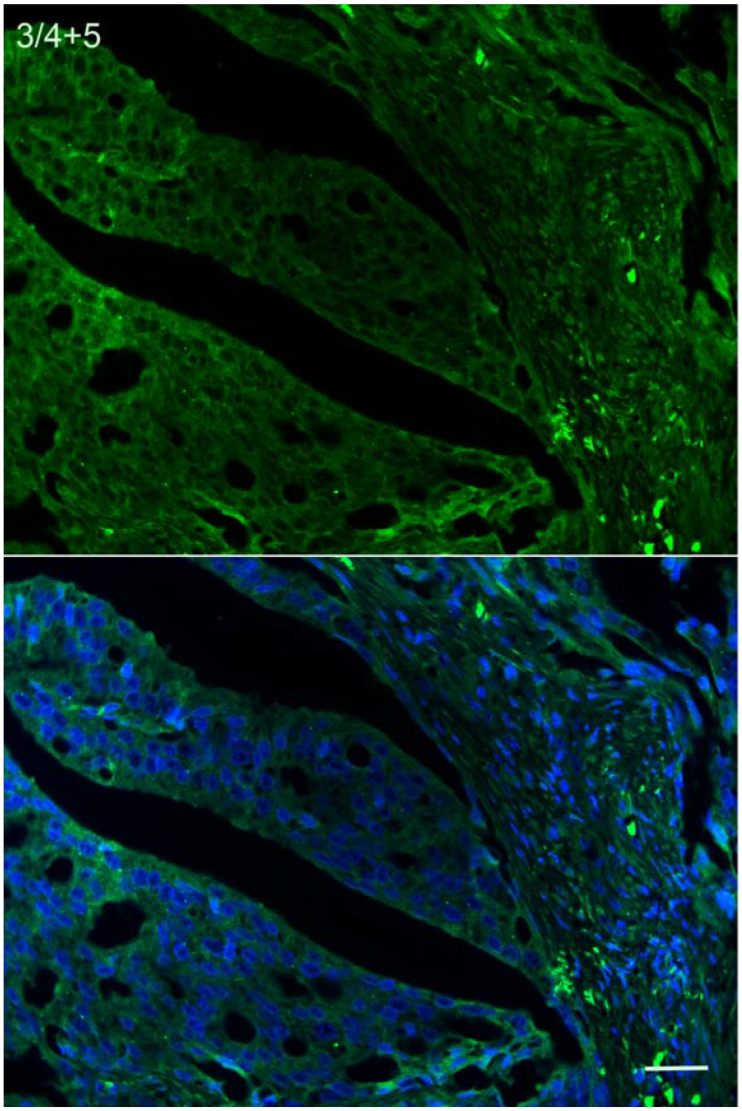

Supplementary Figure 2S: (*Continued*) TRPM8 expression pattern in the prostate cancer patient grade 3, Gleason score 4+5, Related to Figure 1.

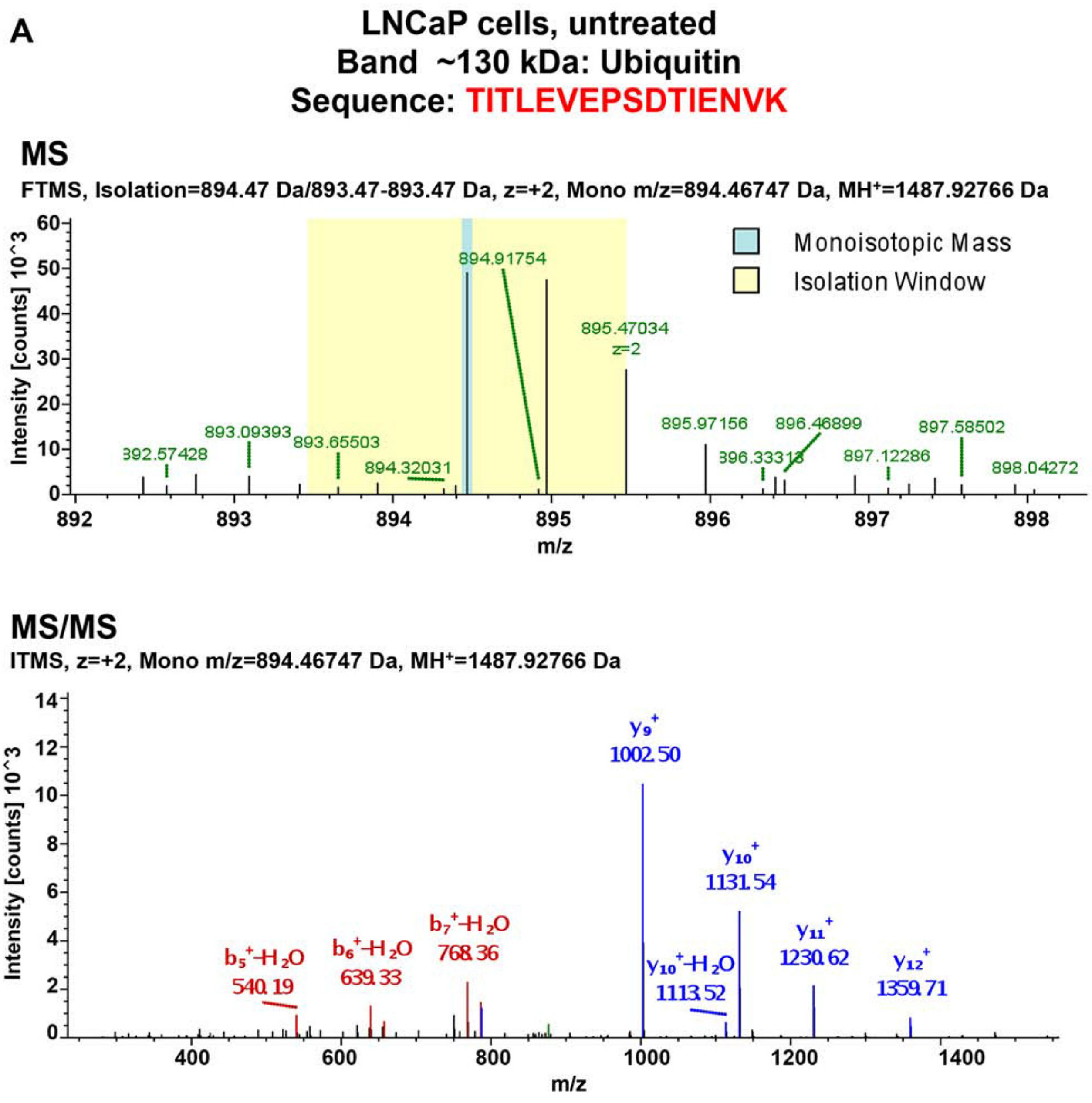

Supplementary Figure 3A: LC-MS and MS/MS analysis of TRPM8 immunoprecipitated from the control LNCaP cells indicates the presence of co-precipitated ubiquitin, Related to Figure 3. (*Continued*)

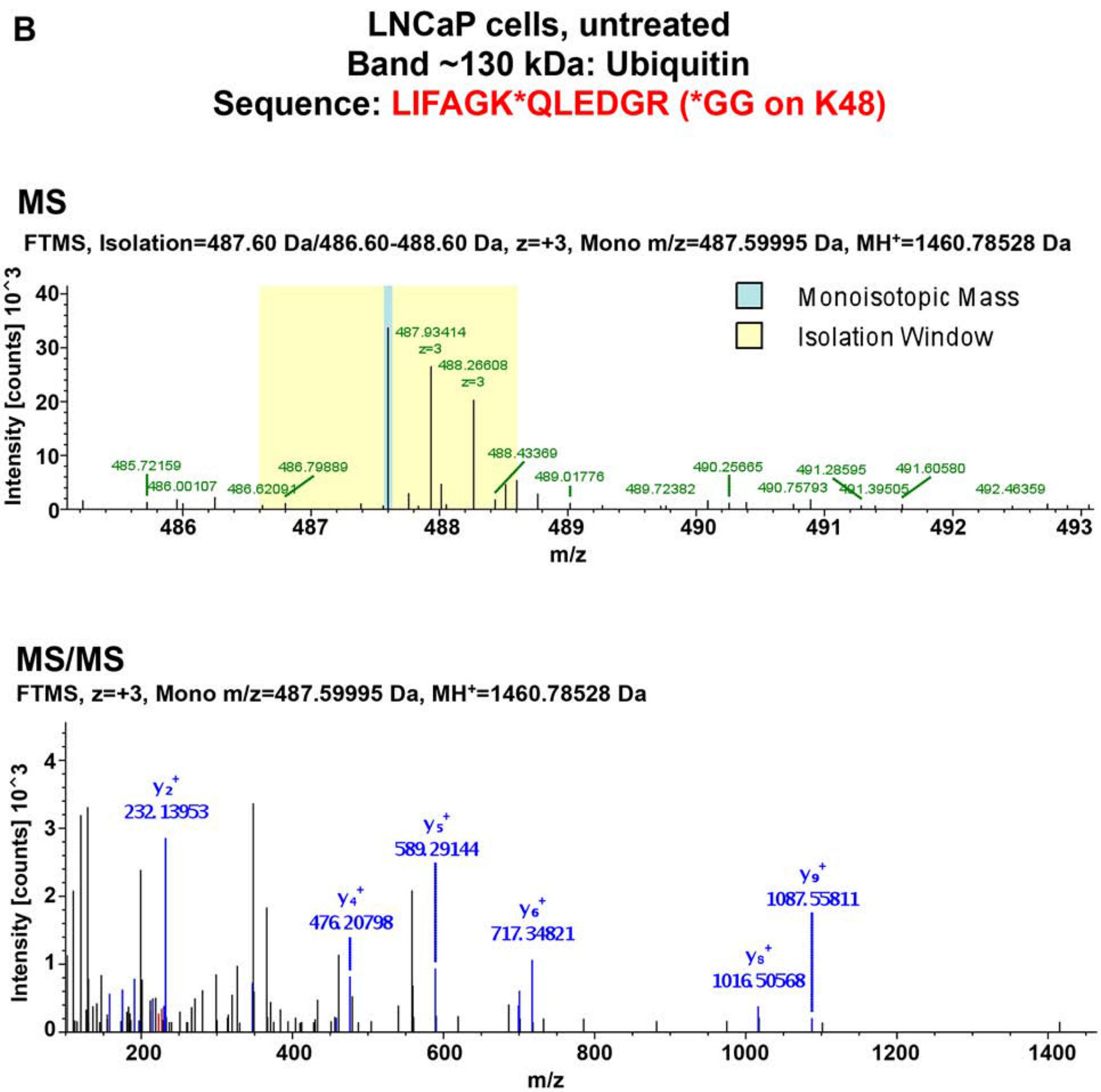

Supplementary Figure 3B: (*Continued*) LC-MS and MS/MS analysis of TRPM8 immunoprecipitated from the control LNCaP cells indicates the presence of co-precipitated polyubiquitin with the K48-linkage, Related to Figure 3.

(*Continued*)

C

LNCaP cells, PYR-41/HF-treated
Band ~130 kDa: TRPM8
Sequence: NDINAAGESEELANEYETR

MS

FTMS, Isolation=1063.47 Da/1062.47-1064.47 Da, z=+2, Mono m/z=1062.97046 Da, MH⁺=2124.933 Da

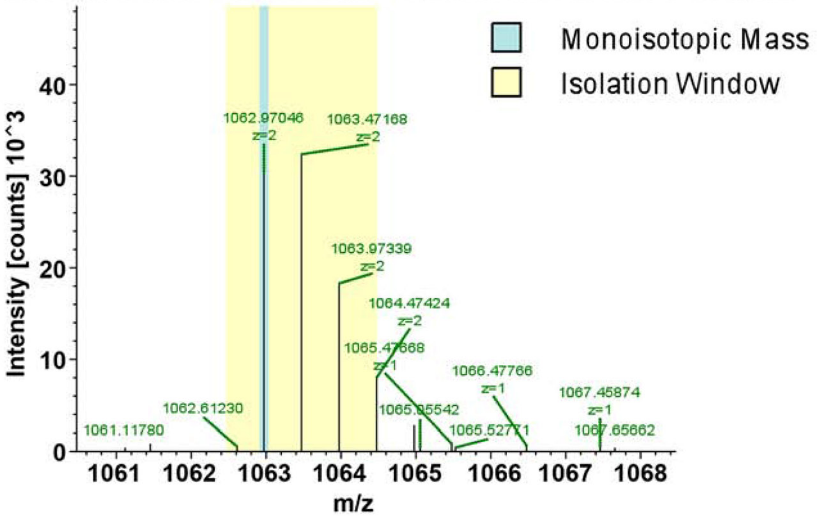

MS/MS

FTMS, z=+2, Mono m/z=1062.97046 Da, MH⁺=2124.933 Da

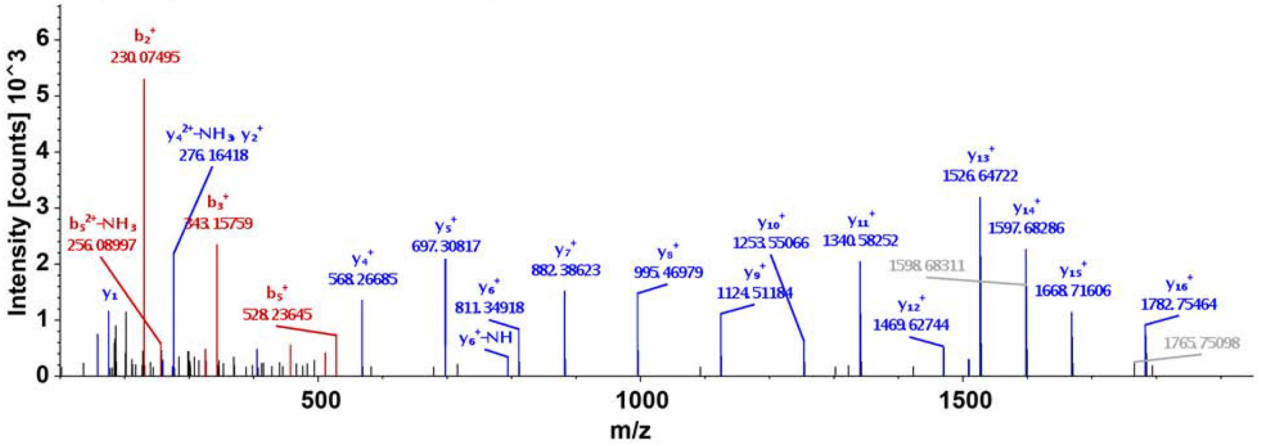

Supplementary Figure 3C: (*Continued*) LC-MS and MS/MS analysis of TRPM8 immunoprecipitated from the PYR-41/HF-treated LNCaP cells indicates recovery of the TRPM8 protein, Related to Figure 3. (*Continued*)

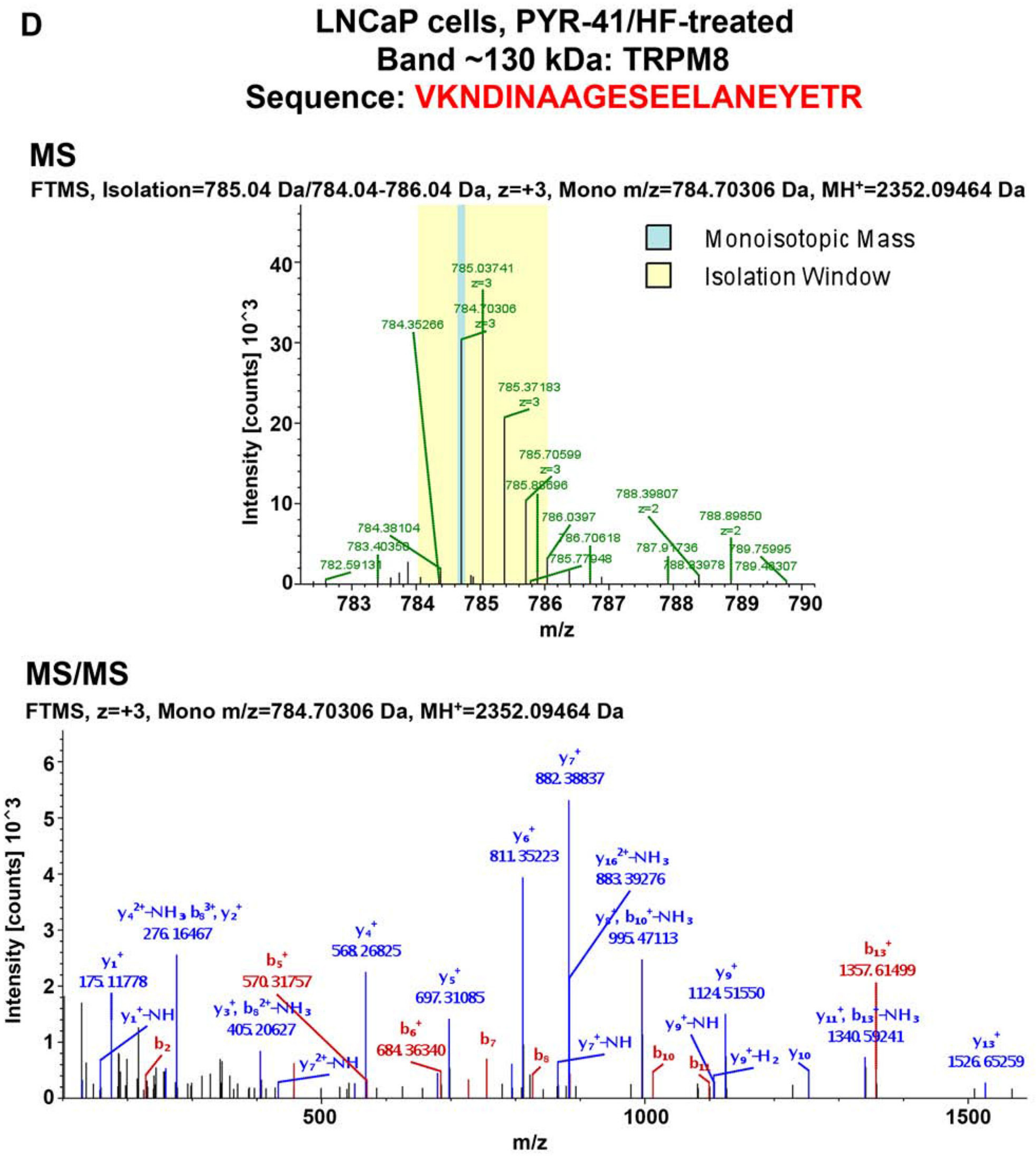

Supplementary Figure 3D: (*Continued*) LC-MS and MS/MS analysis of TRPM8 immunoprecipitated from the PYR-41/HF-treated LNCaP cells indicates recovery of the TRPM8 protein, Related to Figure 3. (*Continued*)

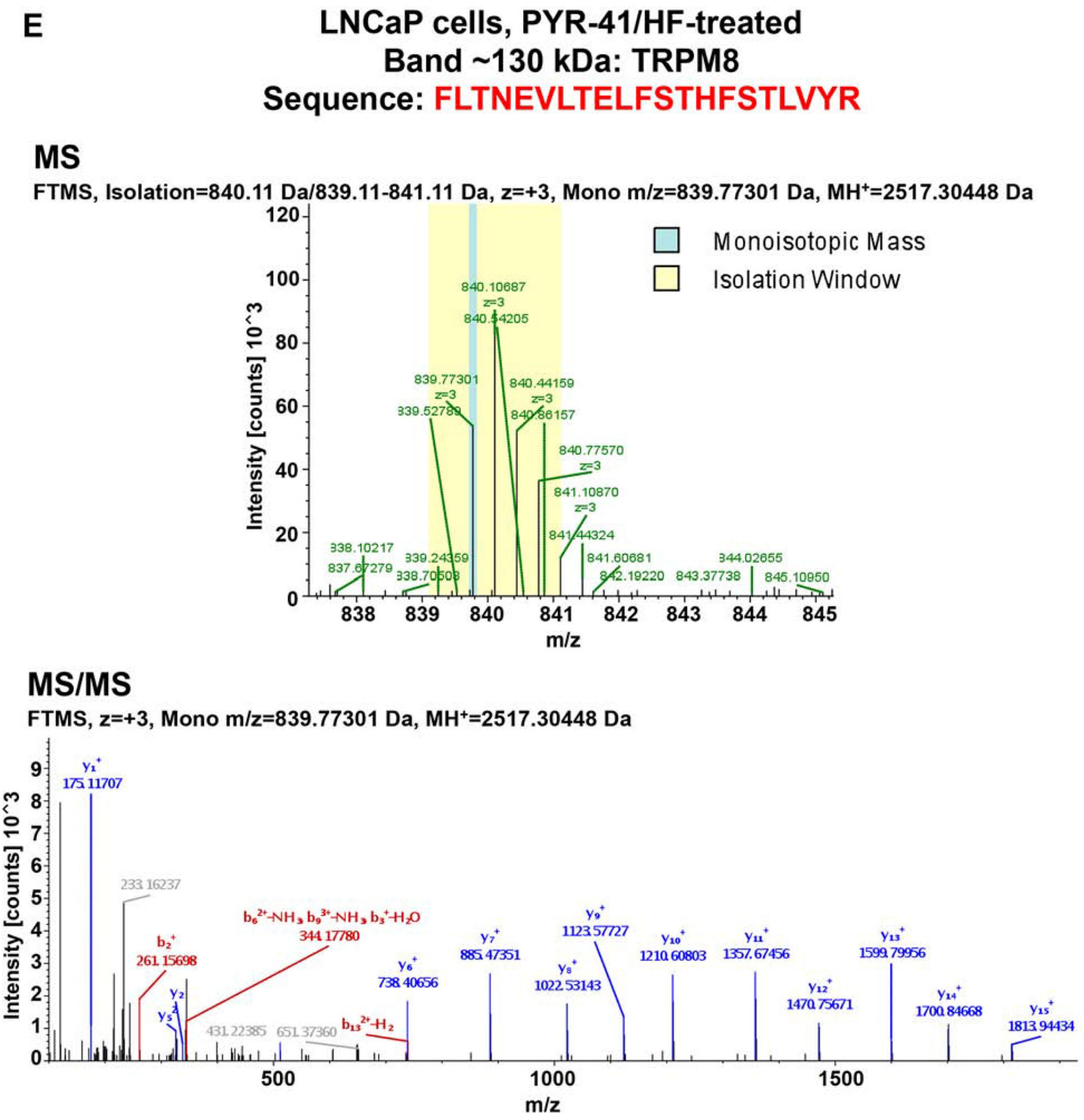

Supplementary Figure 3E: (*Continued*) LC-MS and MS/MS analysis of TRPM8 immunoprecipitated from the PYR-41/HF-treated LNCaP cells indicates recovery of the TRPM8 protein, Related to Figure 3. (*Continued*)

F

LNCaP cells, PYR-41/HF-treated
Band ~130 kDa: TRPM8
Sequence: NEDNETLAWEGVMK

MS

FTMS, Isolation=826.37 Da/827.37-827.37 Da, z=+2, Mono m/z=826.36731 Da, MH⁺=1651.72734 Da

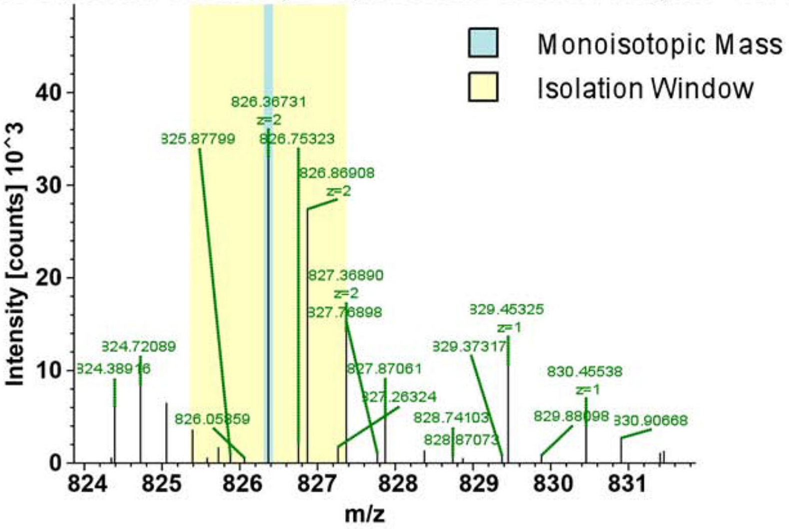

MS/MS

FTMS, z=+2, Mono m/z=826.36731 Da, MH⁺=1651.72734 Da

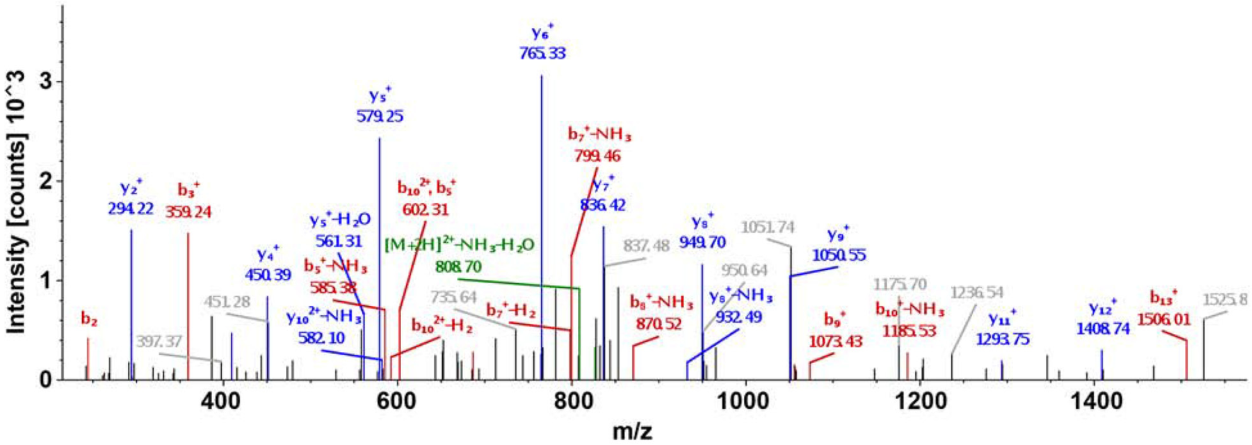

Supplementary Figure 3F: (*Continued*) LC-MS and MS/MS analysis of TRPM8 immunoprecipitated from the PYR-41/HF-treated LNCaP cells indicates recovery of the TRPM8 protein, Related to Figure 3. (*Continued*)

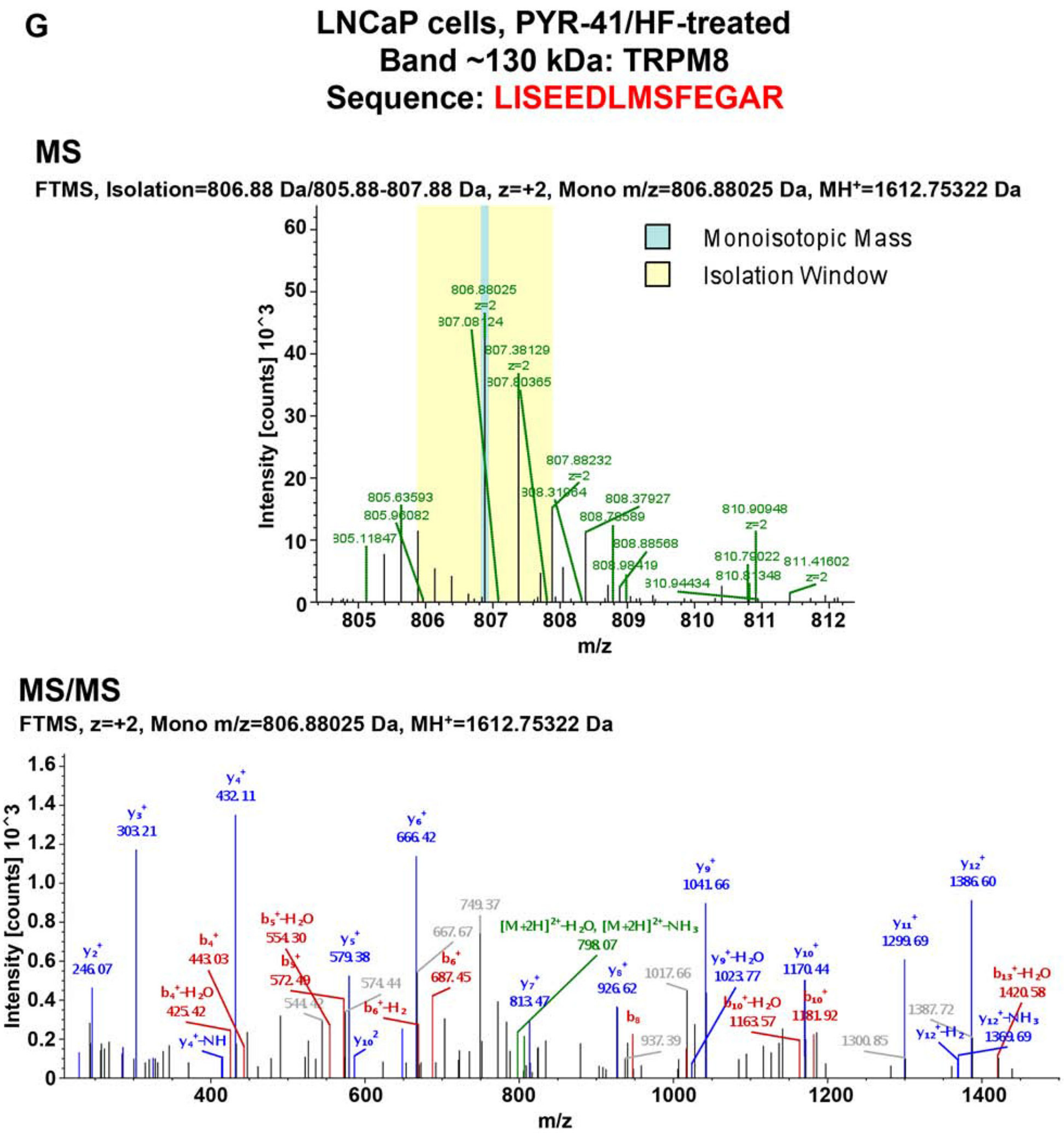

Supplementary Figure 3G: (*Continued*) LC-MS and MS/MS analysis of TRPM8 immunoprecipitated from the PYR-41/HF-treated LNCaP cells indicates recovery of the TRPM8 protein, Related to Figure 3. (*Continued*)

H

LNCaP cells, PYR-41/HF-treated
Band ~130 kDa: TRPM8
Sequence: LIHIFTVSR

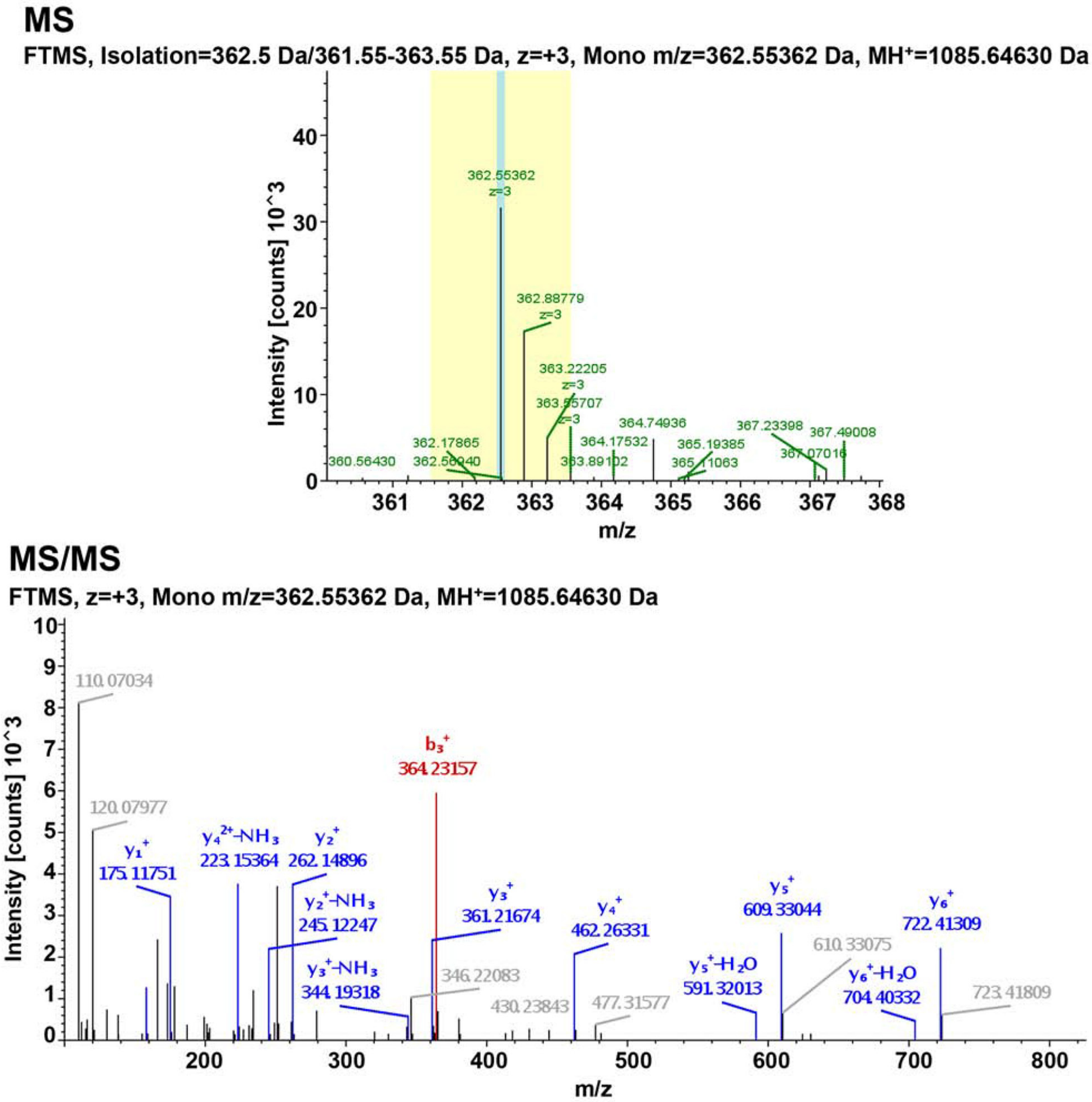

Supplementary Figure 3H: (*Continued*) LC-MS and MS/MS analysis of TRPM8 immunoprecipitated from the PYR-41/HF-treated LNCaP cells indicates recovery of the TRPM8 protein, Related to Figure 3.

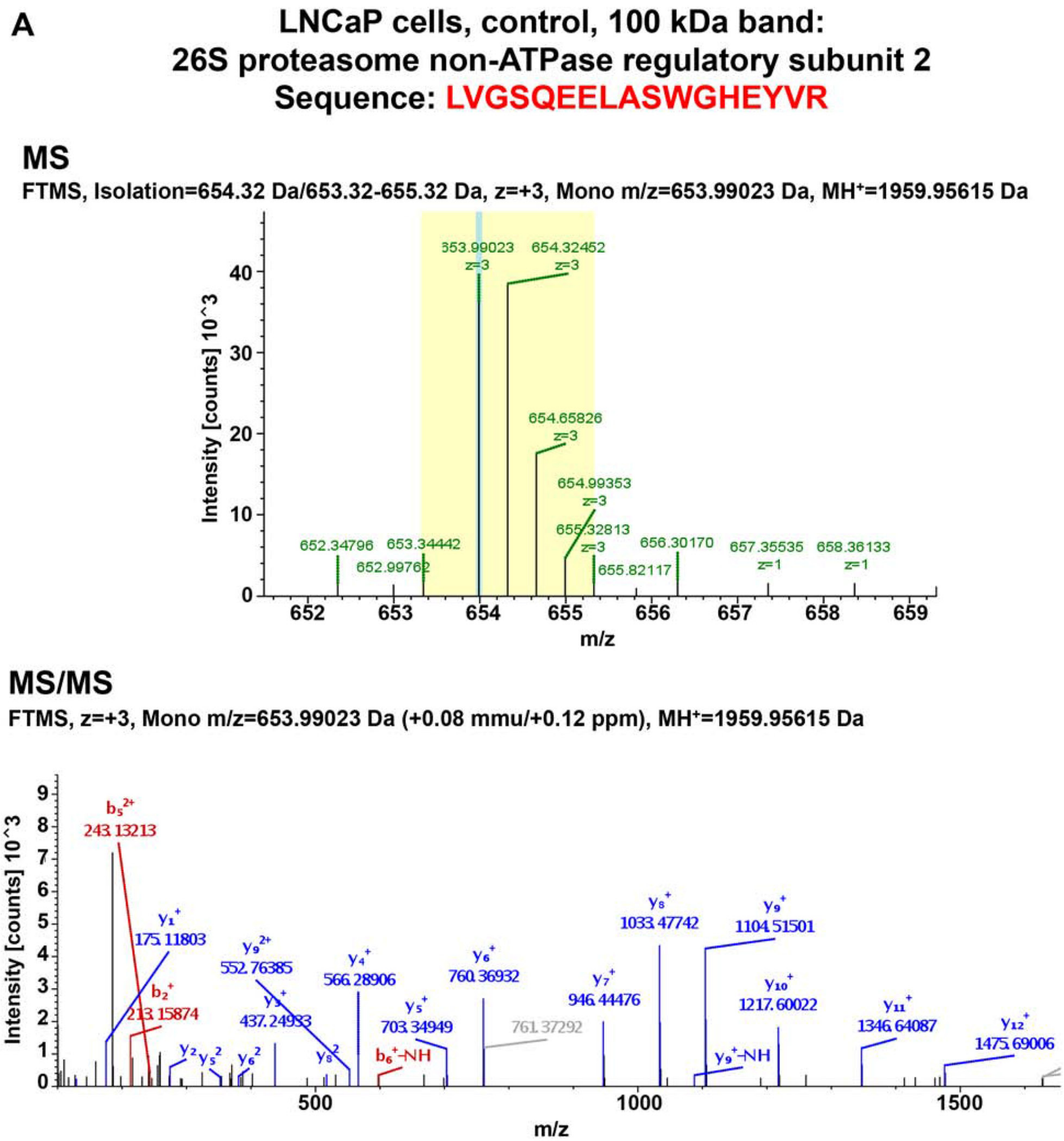

Supplementary Figure 4A: LC-MS and MS/MS analysis of TRPM8 immunoprecipitated from the untreated LNCaP cells indicates the presence of 26S proteasome, Related to Figure 4. (*Continued*)

B

LNCaP cells, control, 100 kDa band: 26S proteasome non-ATPase regulatory subunit 2 Sequence: LNILDTLSK

MS

FTMS, Isolation=508.80 Da/507.80-509.80 Da, z=+3, Mono m/z=508.80353 Da, MH+=508.80353 Da

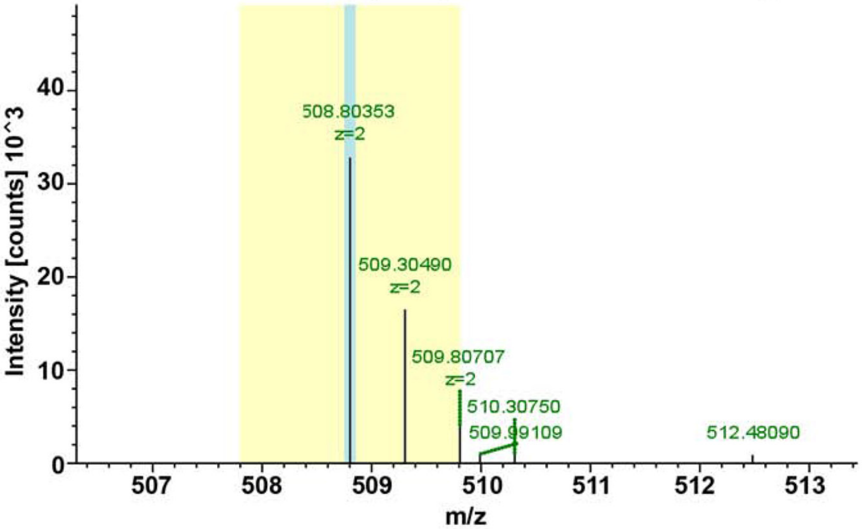

MS/MS

FTMS, z=+3, Mono m/z=508.80353 Da (+0.57 mmu/+1.12 ppm), MH+=508.80353 Da

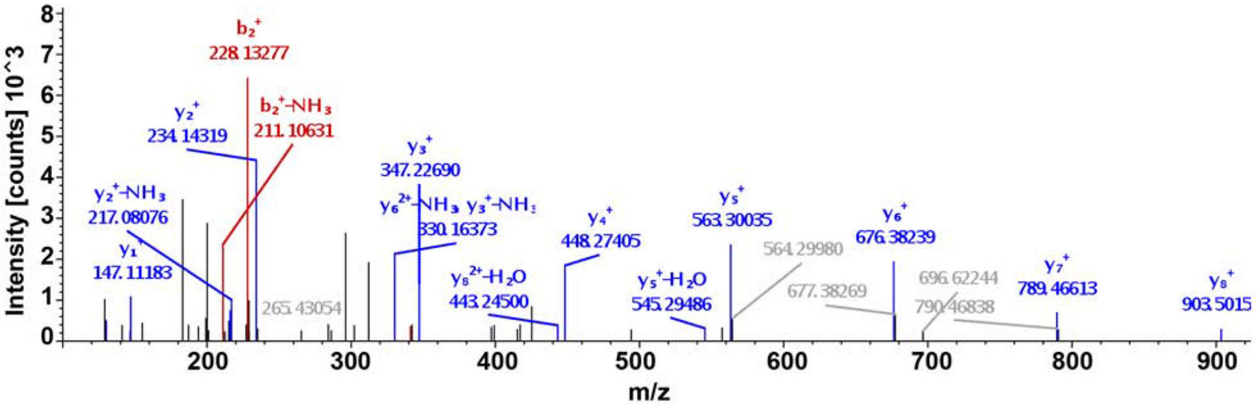

Supplementary Figure 4B: (*Continued*) LC-MS and MS/MS analysis of TRPM8 immunoprecipitated from the untreated LNCaP cells indicates the presence of 26S proteasome, Related to Figure 4. (*Continued*)

C

LNCaP cells, control, 100 kDa band:
Ubiquitin-associated protein 1, UBA1
Sequence: SLSFPKLDSDDSNQKTAK

MS

FTMS, Isolation=776.06 Da/775.06-777.06 Da, z=+3, Mono m/z=775.72107 Da, MH⁺=2325.14865 Da

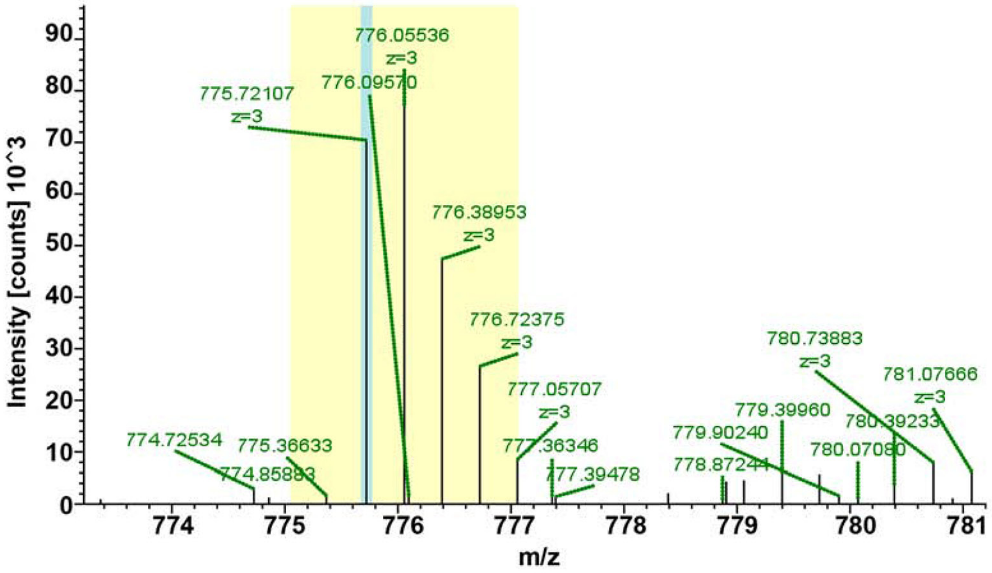

MS/MS

FTMS, z=+3, Mono m/z=775.72107 Da (+4.76 mmu/+6.13 ppm), MH⁺=2325.14865 Da

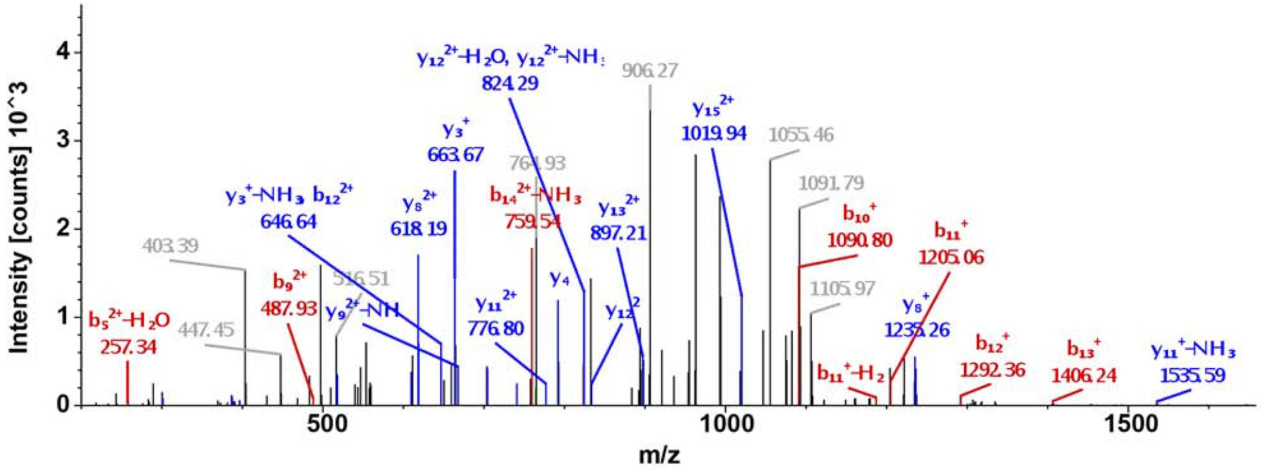

Supplementary Figure 4C: (*Continued*) LC-MS and MS/MS analysis of TRPM8 immunoprecipitated from the untreated LNCaP cells indicates the presence of UBA1, Related to Figure 4. (*Continued*)

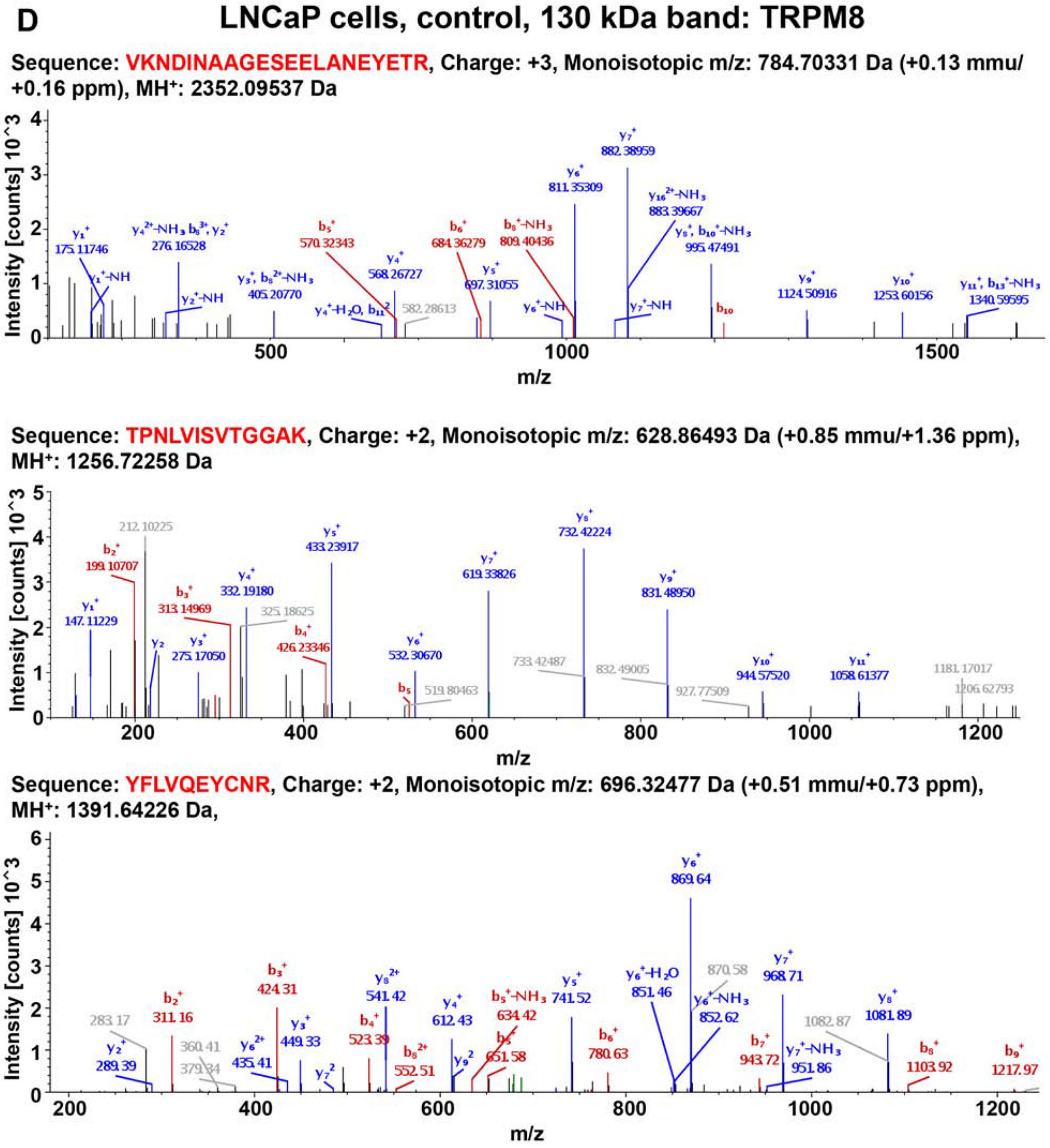

Supplementary Figure 4D: (*Continued*) LC-MS and MS/MS analysis of TRPM8 immunoprecipitated from the untreated LNCaP cells, Related to Figure 4. (*Continued*)

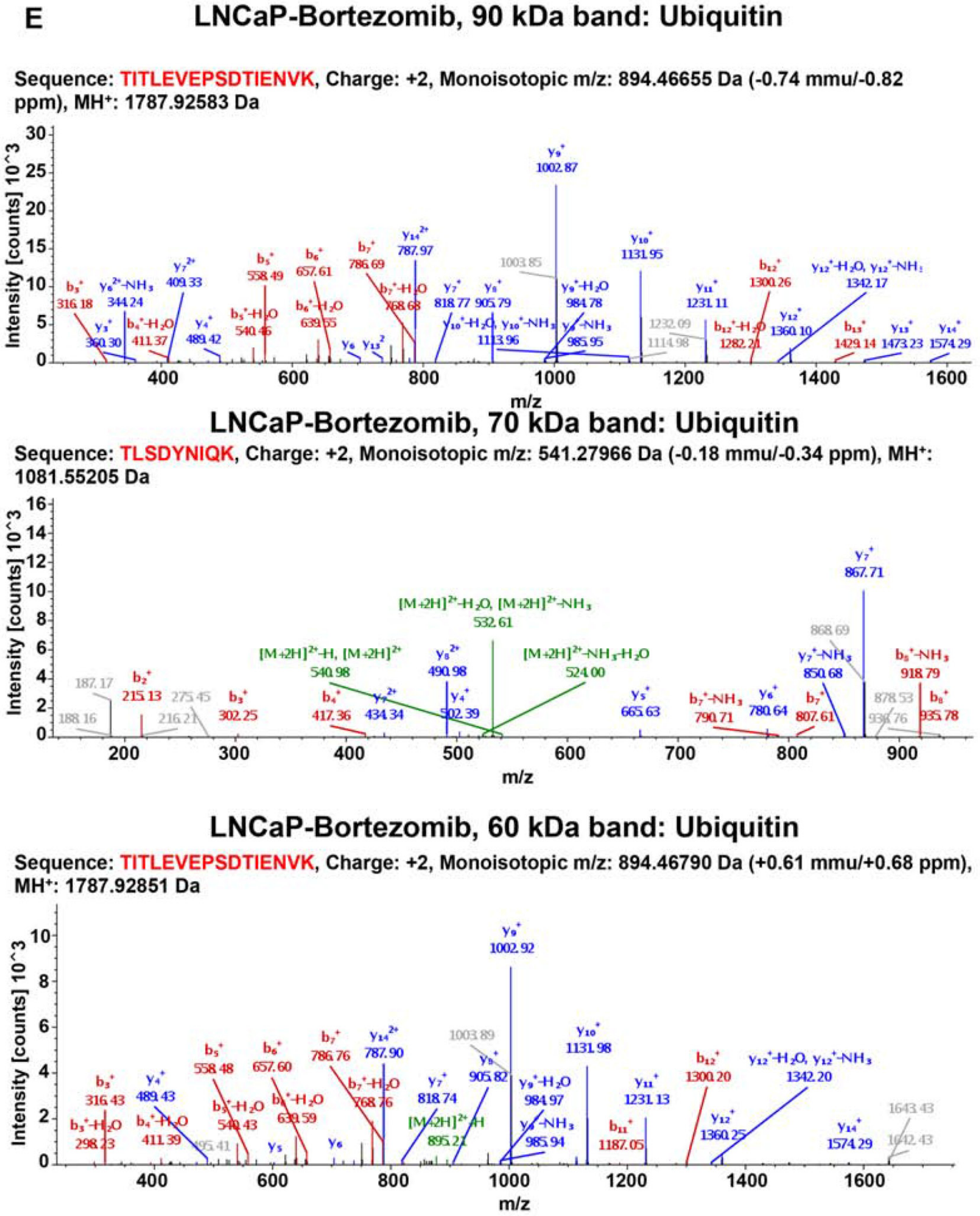

Supplementary Figure 4E: (*Continued*) LC-MS and MS/MS analysis of TRPM8 immunoprecipitated from the bortezomib-treated LNCaP cells, Related to Figure 4. (*Continued*)

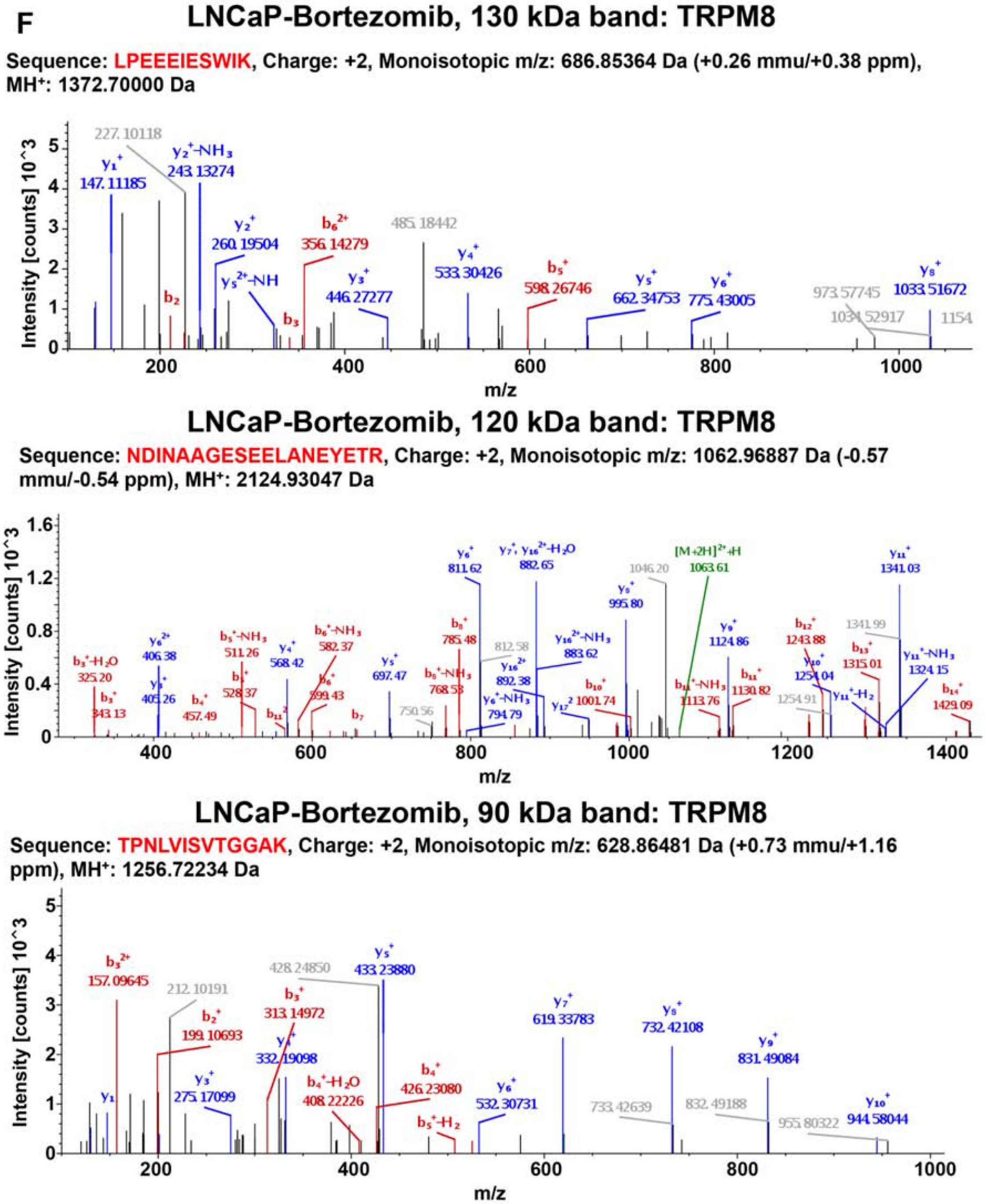

Supplementary Figure 4F: (*Continued*) LC-MS and MS/MS analysis of TRPM8 immunoprecipitated from the bortezomib-treated LNCaP cells, Related to Figure 4. (*Continued*)

G

LNCaP-Chloroquine, 85 kDa band: 26S proteasome non-ATPase regulatory subunit 2

Sequence: FGGSGSQVDSAR, Charge: +2, Monoisotopic m/z: 584.27301 Da (-0.07 mmu/-0.13 ppm), MH+: 1167.53874 Da

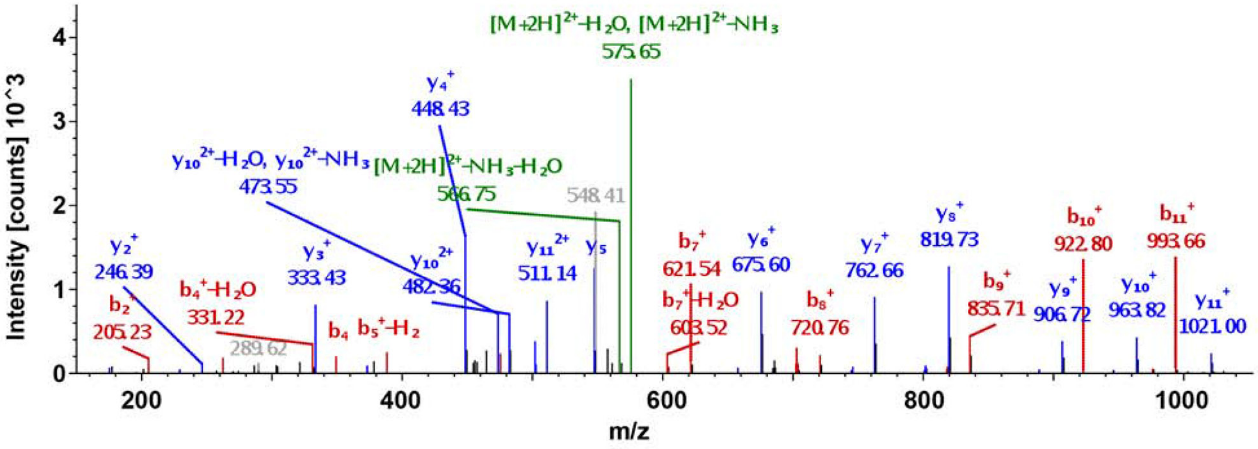

Sequence: LNILDTLSK, Charge: +2, Monoisotopic m/z: 508.80203 Da (-0.93 mmu/-1.82 ppm), MH+: 1016.59679 Da

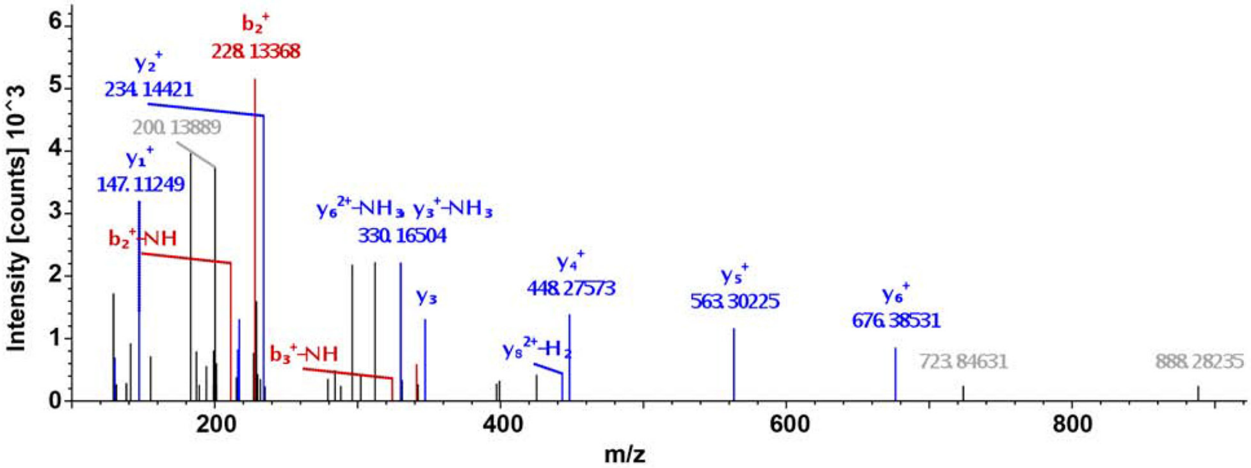

Supplementary Figure 4G: (*Continued*) LC-MS and MS/MS analysis of TRPM8 immunoprecipitated from the chloroquine-treated LNCaP cells, Related to Figure 4. (*Continued*)

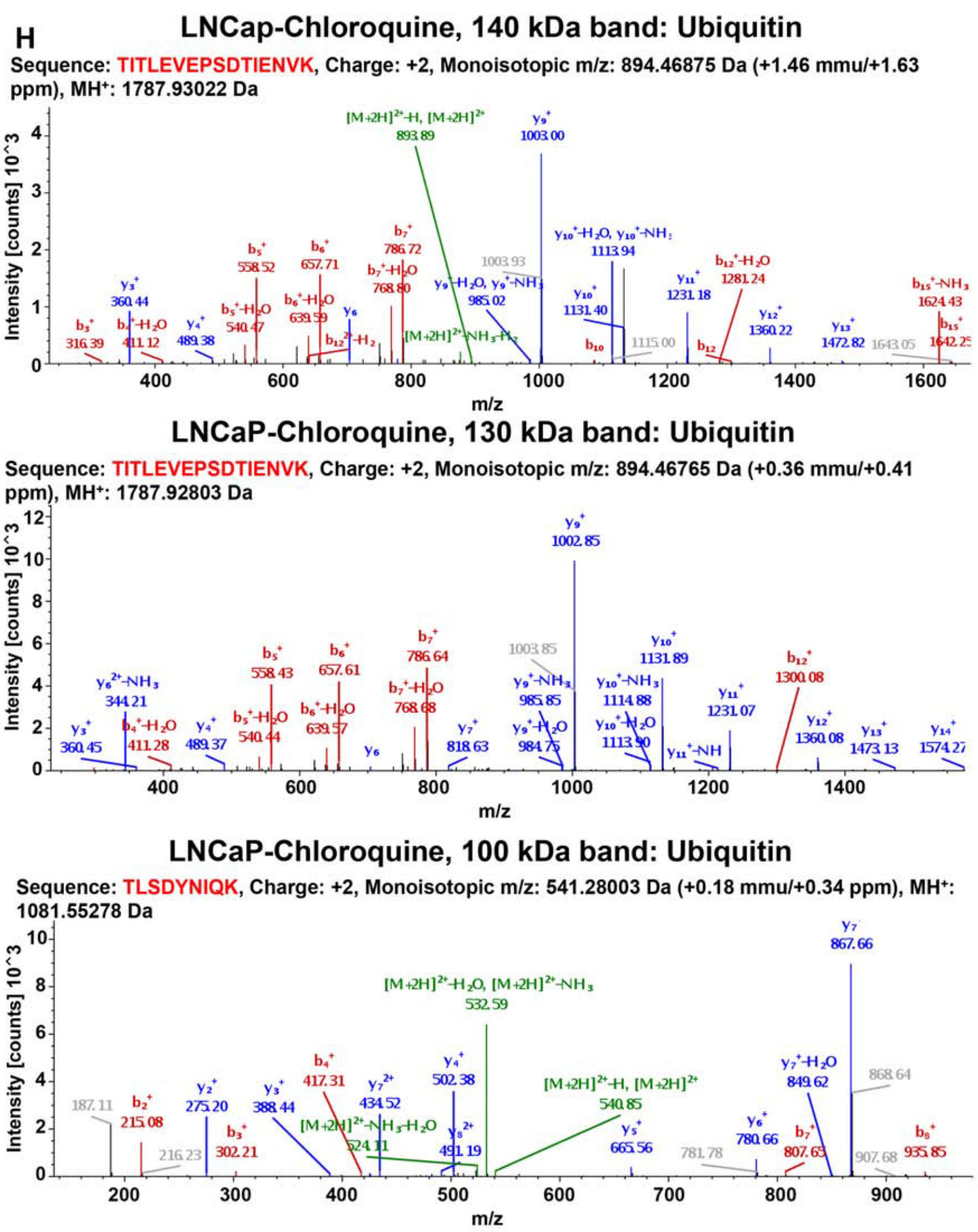

Supplementary Figure 4H: (*Continued*) LC-MS and MS/MS analysis of TRPM8 immunoprecipitated from the chloroquine-treated LNCaP cells, Related to Figure 4. (*Continued*)

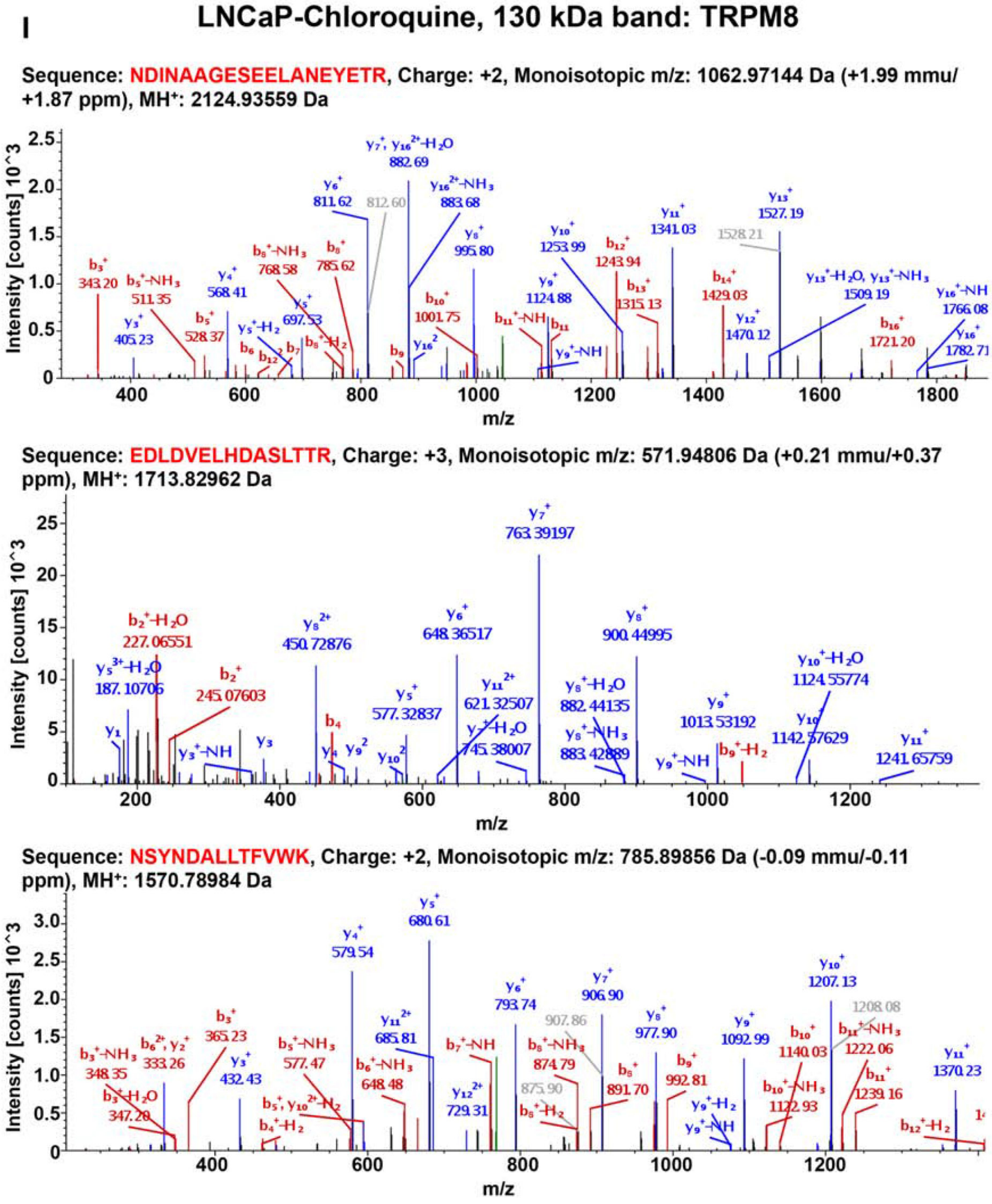

Supplementary Figure 4I: (*Continued*) LC-MS and MS/MS analysis of TRPM8 immunoprecipitated from the chloroquine-treated LNCaP cells, Related to Figure 4.
